# *ADGRV1* Variants in Febrile Seizures/Epilepsy With Antecedent Febrile Seizures and Their Associations With Audio-Visual Abnormalities

**DOI:** 10.3389/fnmol.2022.864074

**Published:** 2022-06-23

**Authors:** Peng Zhou, Heng Meng, Xiaoyu Liang, Xiaoyun Lei, Jingwen Zhang, Wenjun Bian, Na He, Zhijian Lin, Xingwang Song, Weiwen Zhu, Bin Hu, Bingmei Li, Limin Yan, Bin Tang, Tao Su, Hankui Liu, Yong Mao, Qiongxiang Zhai, Yonghong Yi

**Affiliations:** ^1^Key Laboratory of Neurogenetics and Channelopathies of Guangdong Province and the Ministry of Education of China, Department of Neurology, Institute of Neuroscience, The Second Affiliated Hospital, Guangzhou Medical University, Guangzhou, China; ^2^Department of Neurology, The First Affiliated Hospital of Jinan University, Clinical Neuroscience Institute of Jinan University, Guangzhou, China; ^3^Department of Pediatrics, Guangdong Provincial People's Hospital, Guangdong Academy of Medical Sciences, Guangzhou, China; ^4^Department of Neurology, Affiliated Hospital of Putian University, Putian, China; ^5^Department of Neurology, The Second Affiliated Hospital of Hainan Medical University, Haikou, China; ^6^BGI-Shenzhen, Shenzhen, China

**Keywords:** *ADGRV1*, febrile seizures, audio-visual disorders, genotype-phenotype correlation, submolecular effect

## Abstract

**Objective:**

*ADGRV1* gene encodes adhesion G protein-coupled receptor-V1 that is involved in synaptic function. *ADGRV1* mutations are associated with audio-visual disorders. Although previous experimental studies suggested that *ADGRV1* variants were associated with epilepsy, clinical evidence is limited and the phenotype spectrum is to be defined.

**Methods:**

Trio-based targeting sequencing was performed in a cohort of 101 cases with febrile seizure (FS) and epilepsy with antecedent FS. Protein modeling was used to assess the damaging effects of variants. The genotype-phenotype correlations of the *ADGRV1* variants in epilepsy and audio-visual disorders were analyzed.

**Results:**

*ADGRV1* variants were identified in nine unrelated cases (8.91%), including two heterozygous frameshift variants, six heterozygous missense variants, and a pair of compound heterozygous variants. These variants presented a statistically higher frequency in this cohort than that in control populations. Most missense variants were located at CalX-β motifs and changed the hydrogen bonds. These variants were inherited from the asymptomatic parents, indicating an incomplete penetrance. We also identified *SCN1A* variants in 25 unrelated cases (24.75%) and *SCN9A* variants in 3 unrelated cases (2.97%) in this cohort. Contrary to *SCN1A* variant-associated epilepsy that revealed seizure was aggravated by sodium channel blockers, *ADGRV1* variants were associated with mild epilepsy with favorable responses to antiepileptic drugs. The patients denied problems with audio-visual-vestibular abilities in daily life. However, audio-visual tests revealed auditory and visual impairment in the patient with compound heterozygous variants, auditory or vestibular impairment in the patients with heterozygous frameshift, or hydrogen-bond changed missense variants but no abnormalities in the patients with missense variants without hydrogen-bond changes. Previously reported *ADGRV1* variants that were associated with audio-visual disorders were mostly biallelic/destructive variants, which were significantly more frequent in the severe phenotype of audio-visual disorders (Usher syndrome 2) than in other mild phenotypes. In contrast, the variants identified in epilepsy were monoallelic, missense mainly located at CalX-β, or affected isoforms VLGR1b/1c.

**Significance:**

*ADGRV1* is potentially associated with FS-related epilepsy as a susceptibility gene. The genotype, submolecular implication, isoforms, and damaging severity of the variants explained the phenotypical variations. *ADGRV1* variant-associated FS/epilepsy presented favorable responses to antiepileptic drugs, implying a clinical significance.

## Introduction

The human adhesion G protein-coupled receptor V1 (*ADGRV1*) gene (OMIM: 602,851) encodes a very large G protein-coupled receptor-1 (VLGR1), which is localized at synaptic junctions and acts in concert to regulate synaptic function (Neubig and Siderovski, 2002; Togashi et al., 2002). It has also been termed the monogenic audiogenic seizures-susceptibility 1 (*MASS1*) gene, G protein-coupled receptor 98 (*GPR98*) gene, or *VLGR1* gene. Three VLGR1 mRNA isoforms, namely VLGR1a, VLGR1b, and VLGR1c, are expressed in the brain, cochlea, eyes, and connective tissues. VLGR1b, the largest full-length isoform, has a large extracellular domain, encompassing a signal peptide, seven epilepsy-associated repeats (i.e., epitempin repeats), and 35 calcium exchanger β (CalX-β) motifs (Beckmann et al., 1998; Scheel et al., 2002; Staub et al., 2002; Pons et al., 2003; McMillan and White, 2010). Its cytoplasmic domain contains a class I PDZ (i.e., PSD95, Dlg, and ZO-1/ZO-2) binding motif, which is recognized as a ligand for several proteins and is involved in maintaining the structural integrity of hair bundles in the inner ears (Sun et al., 2013). Variants in *ADGRV1* gene are associated with audio-visual disorders, typically Usher syndrome type 2 (USH2), which is characterized by moderate-to-severe congenital sensorineural hearing loss and postnatal retinitis pigmentosa.

Previous studies suggested a potential association between *ADGRV1* gene and epilepsy. The seven epitempin repeats were first identified in the leucine-rich glioma-inactivated 1 gene (Staub et al., 2002), which is associated with autosomal dominant lateral temporal lobe epilepsy with auditory features (Kalachikov et al., 2002). Experimental studies have demonstrated associations between the *Adgrv1/Mass1/Vlgr1* genes and audiogenic seizures in mice. A truncating variant (c.7009delG) of the *Mass1* gene was determined to cause audiogenic seizures in *Frings* mice (Skradski et al., 2001). *Vlgr1*-knockout mice presented a much higher susceptibility to audiogenic seizures (Yagi et al., 2009). Recombinant mutant mice with the deletion of VLGR1 transmembrane and cytoplasmic domains were susceptible to audiogenic seizures (McMillan and White, 2004). A previous study on 48 families with epilepsy with febrile seizures (FSs) identified a nonsense *ADGRV1* variant in a family with two affected siblings, which provided initial clinical evidence on the association between *ADGRV1* and epilepsy (Nakayama et al., 2002). Recent studies have identified ultra-rare *ADGRV1* missense variants in patients with myoclonic epilepsy, FS, genetic generalized epilepsy, and atypical Rolandic epilepsy (Myers et al., 2018; Han et al., 2020; Dahawi et al., 2021; Liu et al., 2022). However, the clinical evidence is generally limited, and the phenotype spectrum of epilepsy is to be defined.

In this study, we screened epilepsy-related genes in 101 unrelated cases with FS or epilepsy with antecedent FS (EFS+) using a targeted sequencing approach and identified eight heterozygous variants and a pair of compound heterozygous variants of *ADGRV1* in nine unrelated cases. The possible impairments of auditory, visual, and vestibular function were also evaluated. We reviewed all reported *ADGRV1* variants and analyzed the correlation between genotype and phenotype, aimed to determine the roles of *ADGRV1* variants in epilepsy and its relationships with audio-visual abnormalities.

## Subjects and Methods

### Subjects

Patients were recruited from the Epilepsy Center of the Second Affiliated Hospital of Guangzhou Medical University and Guangdong Provincial People's Hospital from 2015 to 2021. The cohort consisted of 101 cases with FS-related epilepsy, including 19 cases with FS and 82 cases with EFS+. The detailed clinical information was collected, which contains seizure onset age, seizure type and frequency, course of seizure, response to antiepileptic treatment, family history, and general and neurological examination. Brain magnetic resonance imaging (MRI) scan was conducted to identify structure abnormality. Video electroencephalography (EEG) was performed, and the results were reviewed using two qualified electroencephalographers. Epileptic seizures and epilepsies were diagnosed according to the criteria of the Commission on Classification and Terminology of the ILAE (1981, 1989, 2001, 2010, and 2017). We used the term FS plus (FS+), as in previous reports (Scheffer and Berkovic, 1997; Singh et al., 1999), to denote individuals with FS extending outside the age range definition of 3 months to 6 years or with afebrile generalized tonic-clonic seizures. The activities of the daily life of the patients with *ADGRV1* variants and their parents (asymptomatic carriers) were evaluated to reflect the subjective characteristics of hearing loss, nyctalopia, constriction of the visual fields, and decreased visual acuity. Further audiometric, ophthalmologic, and vestibular tests were performed to detect any subclinical abnormalities. An audiologic evaluation included pure tone audiometry, transient-evoked otoacoustic emission, and auditory brain stem-evoked response recording using the standard protocol. The ophthalmic test included a general ophthalmic examination, Goldmann perimetry, funduscopy, and full-field electroretinography (ERG). The vestibular function was evaluated using infrared video nystagmography, positional nystagmography, and binaural bithermal caloric testing (Smith et al., 1994).

For the controls, whole-exome sequencing (WES) was performed on 296 healthy Chinese volunteers who served as a normal control group as in our previous report (Consortium, 2013). Frequencies of the identified variants were also compared with that in the other control populations, including East Asian and general populations in the Genome Aggregation Database (gnomAD, gnomad.broadinstitute.org) (Karczewski et al., 2020).

This study adhered to the guidelines of the International Committee of Medical Journal Editors with regard to patient consent for research or participation and received approval from the ethics committee of the hospitals. Written informed consents were provided by the patient's legal guardians.

### Targeted Sequencing

All cases were recruited in trios. Blood samples of the probands, their parents, and other available family members were collected to ascertain the source of the variants. Genomic DNA was extracted from blood using the Qiagen Flexi Gene DNA Kit. A gene panel was designed for targeted sequencing on 480 epilepsy-related genes to uncover disease-causing variants (Supplementary Table 1). These genes include 62 epilepsy genes, 34 neurodevelopmental epilepsy genes, 159 epilepsy-related genes, 52 potential epilepsy-associated genes, and 173 genes that are suspected to be related to epilepsy, according to the classification of epilepsy-associated genes (Wang et al., 2017).

Raw read data were aligned on the human assembly genome reference consortium human genome build 37 (GRCh37, also known as hg19) using the Burrows-Wheeler Alignment (Li and Durbin, 2010). The single nucleotide variant and indel calling and filtering were performed using the Genome Analysis Toolkit as previously described (DePristo et al., 2011). According to the guidelines for investigating causality of sequence variants in human disease proposed by the U.S. National Human Genome Research Institute (MacArthur et al., 2014) and the interpretation methods in a previous study (Torkamani et al., 2014), a series of filters were applied to derive a set of candidate disease-causing variants in this study. First, population-based filtration removed variants presenting a minor allele frequency (MAF) ≥0.005 in the Genome Aggregation Database (gnomad.broadinstitute.org), except for those variants previously reported in the Human Gene Variant Database (HGMD) and/or OMIM database. Second, annotation-based filtration removed variants in segmental duplication regions that are prone to produce false-positive variant calls due to mapping errors. Third, functional impact-based filtration retained frame-shift and nonsense variants. Missense variants were included when predicted to be deleterious in sequence conservation or damaging in protein function by one or more *in silico* tools (http://varcards.biols.ac.cn/). Splice-site variants were included when predicted to have altered splicing using the Human Splicing Finder. Fourth, phenotype-based filtration retained variants based on clinical concordance between the phenotypes of patients and previously reported phenotypes of the mutated genes. Following filtering, Sanger sequencing was employed to validate the potential pathogenic variants. The position number for the variants has been obtained from the start codon, i.e., ATG, of the full-length *ADGRV1* isoform sequence (RefSeq accession number: NG_007083.2).

### Molecular Modeling of VLGR1b

Protein modeling was performed by using the Iterative Threading ASSEmbly Refinement (I-TASSER) (Roy et al., 2010) software to evaluate the damaging effect of the amino acid substitution on the VLGR1b protein structure. The confidence of each model was quantitatively measured by a C-score in the range of [−5,2]. The PyMOL Molecular Graphics System (Version 2.3.2; Schrödinger, LLC; New York, USA) was used for three-dimensional protein structure visualization and analysis.

### Analysis of Genotype-Phenotype Correlation and Statistics

We reviewed all *ADGRV1* variants from the HGMD (http://www.hgmd.cf.ac.uk/ac/index.php) and PubMed (http://www.ncbi.nlm.nih.gov/pubmed/) up to December 2021.

The audio-visual disorders associated with *ADGRV1* variants were classified into clinical subtypes as Usher type 2 (USH2), Usher type 3 (USH3), nonsyndromic hearing loss, and nonsyndromic retinitis pigmentosa based on the representations in the original reports. USH2 is characterized by moderate to severe congenital sensorineural hearing loss and later development of retinitis pigmentosa. USH3 is relatively milder and characterized by postlingual hearing loss and variable retinitis pigmentosa and vestibular dysfunction (Millan et al., 2011).

Variants are generally classified into destructive and missense variants. Destructive variants are referred to as those causing gross protein malformations and haploinsufficiency, including truncating variants (i.e., nonsense and frameshifting), splice-site variants, and variants with genomic rearrangement (Wei et al., 2017).

The statistical analysis was performed using SPSS version 22.0 (SPSS Inc., Chicago, IL). The frequencies of the *ADGRV1* variants between the epilepsy cohort and the controls were compared by a two-sided Fisher's exact test. Fisher's exact test and the chi-square test were used to analyze the variants between epilepsy and audio-visual disorders. Values of *p* < 0.05 (two-sided) were considered significant.

## Results

### ADGRV1 Variants

**Figure 1:**
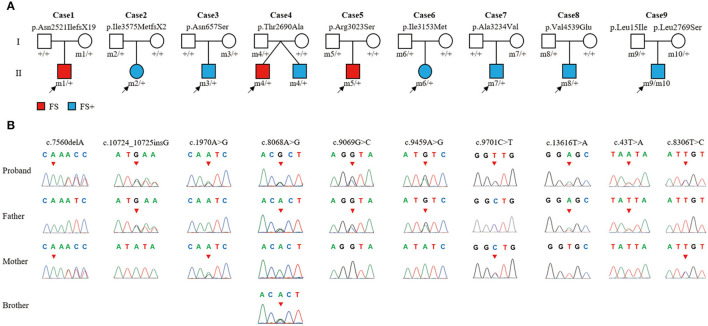
The *ADGRV1* variants identified in the nine cases of febrile seizures or epilepsy with antecedent febrile seizures. **(A)** Pedigrees of the nine cases with *ADGRV1* variants and their corresponding phenotypes. **(B)** DNA sequence chromatograms of the *ADGRV1* variants. Arrows indicate the positions of the nucleotide changes.

In this cohort, 10 *ADGRV1* variants were identified in 9 unrelated cases with FS or FS+ (Figure 1 and Table 1). *ADGRV1* variants identified in this study included two heterozygous frameshift variants (i.e., c.7560delA/p.Asn2521IlefsX19 and c.10724_10725insG/p.Ile3575MetfsX2), six heterozygous missense variants (i.e., c.1970A>G/p.Asn657Ser, c.8086A>G/p.Thr2690Ala, c.9096G>C/p.Arg3023Ser, c.9459A>G/p.Ile3153Met, c.9701C>T/p.Ala3234Val, and c.13616T>A/p.Val4539Glu), and a pair of compound heterozygous variants (i.e., c.43T>A/p.Leu15Ile and c.8306T>C/p.Leu2769Ser). The variant p.Thr2690Ala was identified in a pair of affected twins (Case 4). These variants were inherited from their asymptomatic parents.

**Table 1:** Clinical features of individuals with *ADGRV1* variants.

**Case**	**Variant (NM_032119)**	**Gender**	**Age**	**FS onset**	**aFS onset**	**Seizure course**	**Seizure-free duration**	**Effective AEDs**	**EEG**	**Diagnosis**
1	c.7560delA (p.Asn2521IlefsX19)	Male	6 yr	3 d	-	GTCS, 2~4 times/mo	5 yr	LTG	Right central spikes or slow waves	FS
2	c.10724_10725insG (p.Ile3575MetfsX2)	Female	11 yr	4 yr	4 yr	GTCS, 1~3 times/yr	5 yr	VPA	Generalized spikes and spike-slow waves	FS+
3	c.1970A>G (p.Asn657Ser)	Male	15 yr	8 mo	10 yr	sGTCS, 1 time/yr	3 yr	VPA	Right temporal sharp-slow waves	FS+
4-1	c.8068A>G (p.Thr2690Ala)	Male	12 yr	2 yr	-	GTCS, 1~2 times/yr	3 yr	-	Normal	FS
4-2		Male	12 yr	2 yr	-	GTCS, 1~2 times/yr	4 yr	-	Normal	FS+
5	c.9069G>C (p.Arg3023Ser)	Male	12 yr	4 yr	-	GTCS, 1 time/yr	6 yr	-	Generalized spikes and spike-slow waves	FS
6	c.9459A>G (p.Ile3153Met)	Female	11 yr	2 yr	-	GTCS, 1~2 times/yr	4 yr	VPA	Generalized 2.5-3.0 Hz spike-slow waves and multiple focal spike-slow waves	FS
7	c.9701C>T (p.Ala3234Val)	Male	9 yr	9 mo	3 yr	GTCS, sGTCS, 1~2 times/yr	4 yr	-	Normal	FS+
8	c.13616T>A (p.Val4539Glu)	Male	11 yr	1 yr	5 yr	GTCS, 1~2 times/yr	3 yr	VPA	Bilateral parietal single sharp waves	FS+
9	c.43T>A (p.Leu15Ile) c.8306T>C (p.Leu2769Ser)	Male	14 yr	5 yr	6 yr	sGTCS, 1~3 times/yr	4 yr	OXC, VPA, LTG	Asymmetric generalized spike-slow waves	FS+

*AEDs, antiepileptic drugs; aFS, afebrile seizures; d, day; EEG, electroencephalogram; FS, febrile seizures; FS+, febrile seizures plus; GTCS, generalized tonic-clonic seizures; LTG, lamotrigine; mo, month; OXC, oxcarbazepine; sGTCS, secondary generalized tonic–clonic seizure; VPA, valproate; yr, year*.

The 8 missense variants were absent or presented as rare (MAF <0.005) in the gnomAD database (Table 2). The aggregate frequency of these variants in this cohort was significantly higher than that in the controls of 296 normal individuals (11/202 vs. 2/592; *p* = 1.10×10^−5^), the gnomAD-all population (vs. 89/203,608; *p* < 2.20×10^−16^), the controls of the gnomAD-all population (vs. 43/81,262; *p* < 2.20×10^−16^), the gnomAD-East Asian population (vs. 87/14,282, *p* = 1.15×10^−7^), and the controls of the gnomAD-East Asian population (vs. 42/6,074, *p* = 6.82×10^−7^).

**Table 2:** Analysis of the aggregate frequency of *ADGRV1* variants identified in this study.

	**Allele count/ number in this study**	**Allele count/number in controls of 296 healthy volunteers**	**Allele count/number in gnomAD-all populations**	**Allele count/number in gnomAD-East Asian**	**Allele count/number in controls of gnomAD-all populations**	**Allele count/number in controls of gnomAD-East Asian populations**
**Identified** ***ADGRV1*** **variants (NM_032119)**						
c.7560delA/p.Asn2521IlefsX19	1/202	-/-	-/-	-/-	-/-	-/-
c.10724_10725insG/p.Ile3575MetfsX2	1/202	-/-	-/-	-/-	-/-	-/-
c.1970A>G/p.Asn657Ser	1/202	-/-	2/247306	0/17910	1/107964	0/8638
c.8068A>G/p.Thr2690Ala	2/202	-/-	13/280314	13/19530	5/119390	5/9552
c.9069G>C/p.Arg3023Ser	1/202	1/592	8/203608	8/14282	4/81262	4/6074
c.9459A>G/p.Ile3153Met	1/202	-/-	-/-	-/-	-/-	-/-
c.9701C>T/p.Ala3234Val	1/202	1/592	52/280398	52/19522	28/119346	28/9546
c.13616T>A/p.Val4539Glu	1/202	-/-	1/247234	1/17890	0/107948	0/8586
c.43T>A/p.Leu15Ile	1/202	-/-	-/-	-/-	-/-	-/-
c.8306T>C/p.Leu2769Ser	1/202	-/-	13/229608	13/16202	5/94586	5/7246
**Total**	11/202 (0.054)	2/592 (0.0034)	89/203608 (0.0025)	87/14282 (0.032)	43/81262 (0.0029)	42/6074 (0.036)
P value^†^		1.10 × 10^−5^	< 2.20 × 10^−16^	1.15 × 10^−7^	< 2.20 × 10^−16^	6.82 × 10^−7^
OR (95% CI)		16.91 (3.65–158.29)	132.13 (62.42–250.90)	9.39 (4.45–18.02)	108.60 (49.78–216.96)	8.26 (3.78–16.65)

† *p-values and odds ratio were estimated with a 2-sided Fisher's exact test*. *CI, confidence interval; gnomAD, Genome Aggregation Database; OR, odds ratio*.

We also identified 25 *SCN1A* variants (including 18 *de novo*) in 25 unrelated cases (24.75%) and 3 *SCN9A* variants in three unrelated cases (2.97%) in this cohort (Supplementary Table 2). We did not detect variants in the other potential FS-associated genes (such as *SCN1B, GABRG2, GABRD*, and *CPA6*) (Wang et al., 2017) in this cohort.

### Clinical Features of Epilepsy

Clinical data of the nine cases with *ADGRV1* variants are shown in Table 1. Onset ages of FS ranged from the third day of life to 5 years. All cases experienced a few febrile or afebrile generalized tonic-clonic seizures or secondarily generalized tonic-clonic seizures per year.

**Figure 2:**
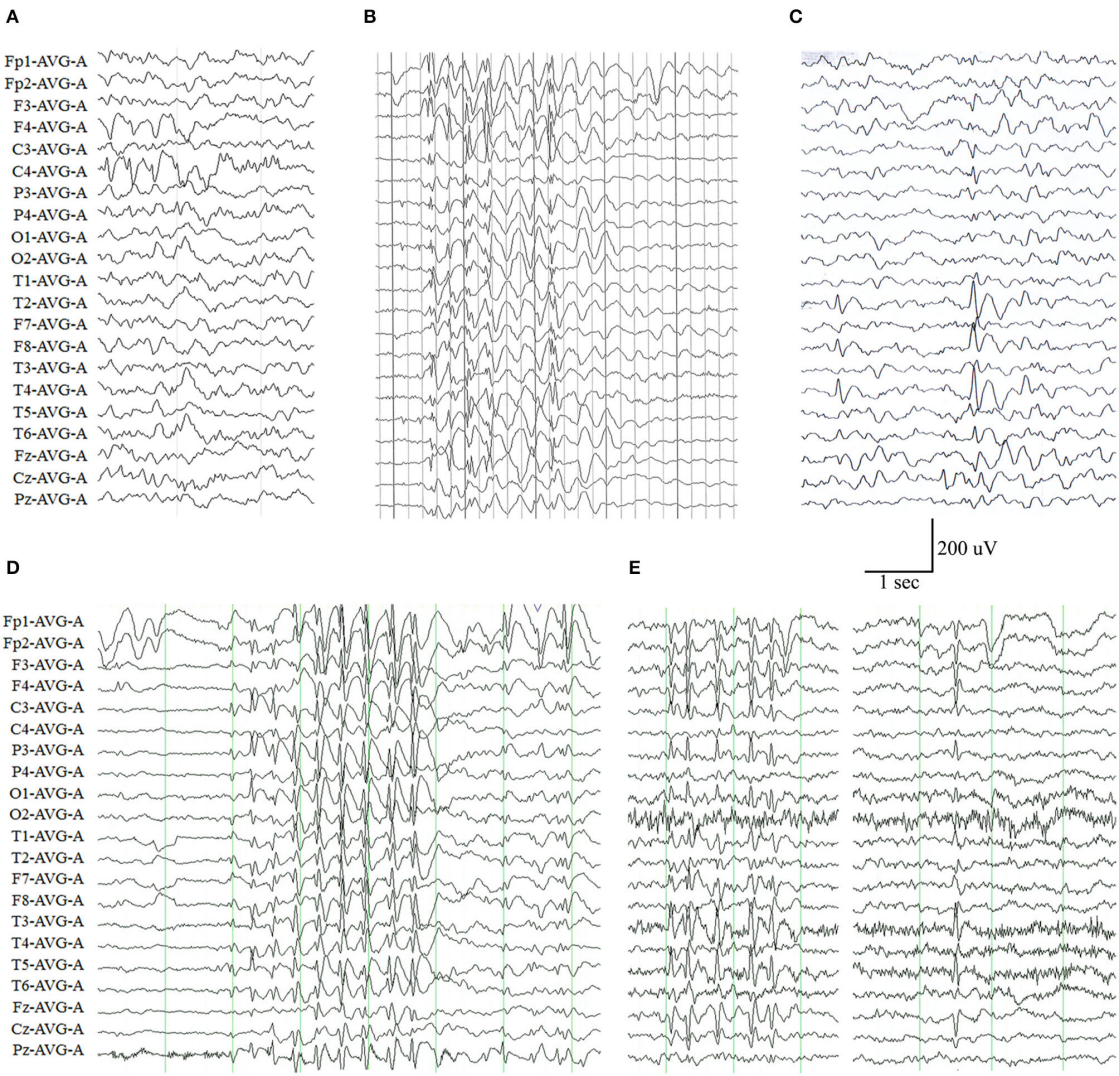
Electroencephalography (EEG) changes in the cases with *ADGRV1* variants. **(A)** Interictal EEG of case 1 at the age of 4 months showed spike-slow waves in the posterior right frontal and central lobes. **(B)** Interictal EEG of case 2 at the age of 6 years showed irregular generalized spike-slow waves. **(C)** Interictal EEG of case 3 at the age of 11 years showed spike-slow waves in the right temporal lobe. **(D)** Interictal EEG of case 6 at the age of 5 years showed asymmetric generalized spike-slow waves. **(E)** Interictal EEG of case 9 at the age of 8 years showed irregular and asymmetric generalized spike-slow waves.

The representative abnormal EEGs of these cases are shown in Figure 2. Initial interictal EEGs were normal in two cases (i.e., cases 4 and 7). A variety of EEG abnormalities was found in the other seven cases. Interictal EEGs of four cases (i.e., cases 2, 5, 6, and 9) showed generalized spike-slow wave discharges, which were less regular or asymmetric in cases 2, 6, and 9 (Figures 2B,D,E). Focal sharp, spike, or spike-slow waves were observed in three cases (i.e., cases 1, 3, and 8). The epileptiform waves of cases 1 and 3 were apparently aggravated during slow sleep (Figures 2A,C).

All cases presented favorable outcomes. Cases 4, 5, and 7 became seizure-free without any antiepileptic treatment. The other five cases with heterozygous variants had been seizure-free on monotherapy of valproate or oxcarbazepine or lamotrigine. The case with compound heterozygous variants also responded well to antiepileptic drugs (AEDs), but the seizures occasionally recurred, induced by fatigue mostly. The EEGs of all cases became normal with the achievement of seizure control until the last follow-up.

### Clinical Features of Audio-Visual Abnormalities

All cases and the unaffected variant carriers denied problems of audio-visual-vestibular abilities in daily life. A total of six cases (i.e., cases 1, 2, 3, 4, 7, and 9) received audiometric, ophthalmologic, and vestibular tests (Table 3). Both cases 4 and 7 did not present any auditory or visual problems. Case 2 revealed mild hearing impairment of the right ear. Case 3 revealed mildly decreased sensitivity of the right horizontal semicircular canal in the caloric test. Case 9, who had compound heterozygous variants, presented both subclinical auditory and visual abnormalities, including mild hearing impairment of the right ear, abnormal visual field, moderately reduced cone function of bilateral eyes, and mildly reduced rod function of the right eye. His father, who carried variant p.Leu15Ile, revealed mildly reduced cone function of bilateral eyes.

**Table 3:** Audio-visual examination of six patients and their variant-carried parent(s).

**Family**		**Variant (NM_032119)**	**Pure tone audiometry**	**Transient evoked otoacoustic emission**	**ABR**	**General ophthalmic examination**	**Fundo-scopy**	**ERG**	**Caloric test**
1	P	c.7560delA (p.Asn2521IlefsX19)	N	N	N	UA	UA	UA	UA
	M	c.7560delA (p.Asn2521IlefsX19)	N	N	N	-	-	-	-
2	P	c.10724_10725insG (p.Ile3575MetfsX2)	R mild abnormal at low frequency	N	N	N	N	-	N
	F	c.10724_10725insG (p.Ile3575MetfsX2)	-	-	-	-	-	-	-
3	P	c.1970 A>G (p.Asn657Ser)	N	N	N	N	N	N	R horizontal semicircular canal weakness
	M	c.1970 A>G (p.Asn657Ser)	-	-	-	-	-	-	-
4	P	c.8068A>G (p.Thr2690Ala)	N	N	N	N	N	N	-
	B	c.8068A>G (p.Thr2690Ala)	N	N	N	N	N	N	-
	F	c.8068A>G (p.Thr2690Ala)	N	N	N	-	-	-	-
7	P	c.9701C>T (p.Ala3234Val)	N	N	N	N	N	N	-
	F	c.9701C>T (p.Ala3234Val)	N	N	N	N	N	N	-
9	P	c.43T>A (p.Leu15Ile)	R mild abnormal at low frequency	N	N	N	N	Mild-moderate bilateral Cone/R rod degeneration	N
	F	c.8306T>C (p.Leu2769Ser)	N	N	N	N	N	Mild bilateral Cone	N
	M	c.43T>A (p.Leu15Ile) c.8306T>C (p.Leu2769Ser)	N	N	N	N	N	N	N

*ABR, auditory brain stem evoked response; B, brother; ERG, full-field electroretinography; F, father; M, mother; N, normal; P, proband; R, right; UA, unavailable (for the age was too young to do the test)*.

### Molecular Alteration of VLGR1

Variants p.Asn2521IlefsX19 and p.Ile3575MetfsX2 resulted in frame shifting and premature termination codons. The two mutants were expected to lack not only the functional domains of epitempin and CalX-β but also the entire membrane-spanning region. Among the 8 missense variants, p.Leu15Ile was located in the signal peptide, and p.Ala3234Val was located in epilepsy-associated repeat 1. The remaining six variants were located in CalX-β domains, namely p.Asn657Ser (CalX-β 5), p.Thr2690Ala (CalX-β 19), p.Leu2769Ser (CalX-β 19), p.Arg3023Ser (CalX-β 21), p.Ile3153Met (CalX-β 22), and p.Val4539Glu (CalX-β 31) (Supplementary Table 3). In short, the majority of these missense variants potentially affect the function of CalX-β domains.

**Figure 3:**
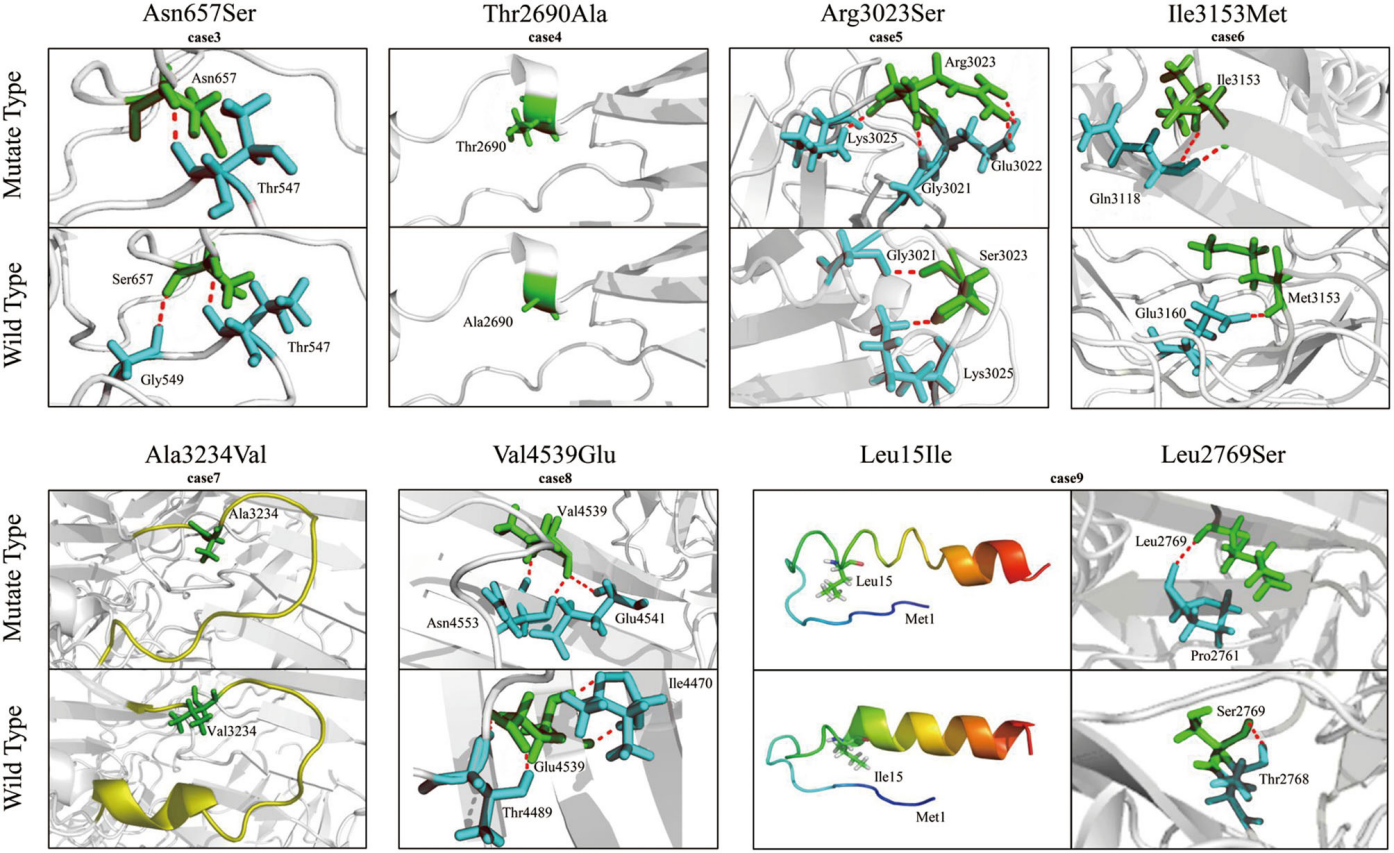
Molecular modeling of VLGR1b and the mutants. Hydrogen bonds were changed in four heterozygous missense variants, namely, Asn657Ser, Arg3023Ser, Ile3153Met, and Val4539Glu. Mutant Thr2690Ala and Ala3234Val did not change any hydrogen bond. Among the compound heterozygous variants, Leu15 is located near an α-helix structure of the signal peptide, and the α-helix structure was extended in the L15I mutant. A new hydrogen bond was formed in the mutant Leu2769Ser. The hydrogen bonds are indicated by dotted red lines.

To evaluate the potentially damaging effect of the missense variants, protein modeling was performed to analyze the protein structure affected by the substitutions (Figure 3). Variants p.Thr2690Ala (from case 4) and p.Ala3234Val (from case 7) did not change their hydrogen bonds. Both cases 4 and 7 presented normal EEGs, were seizure-free without any treatment, and had a normal audio-visual function. Four variants, namely, p.Asn657Ser, p.Arg3023Ser, p.Ile3153Met, and p.Val4539Glu, resulted in new hydrogen bonds with surrounding amino acid residues. Among the compound heterozygous variants, p.Leu15Ile resulted in an extended α-helix structure of the signal peptide, and p.Leu2769Ser formed a new hydrogen bond.

### Genotype-Phenotype Correlation

To date, a total of 268 variants in 155 unrelated cases have been reported (Supplementary Table 3). Most variants have been identified in patients with audio-visual disorders (240 variants in 130 cases); additional 28 variants have been identified in 25 cases with epilepsy, including 10 variants in this study.

**Figure 4:**
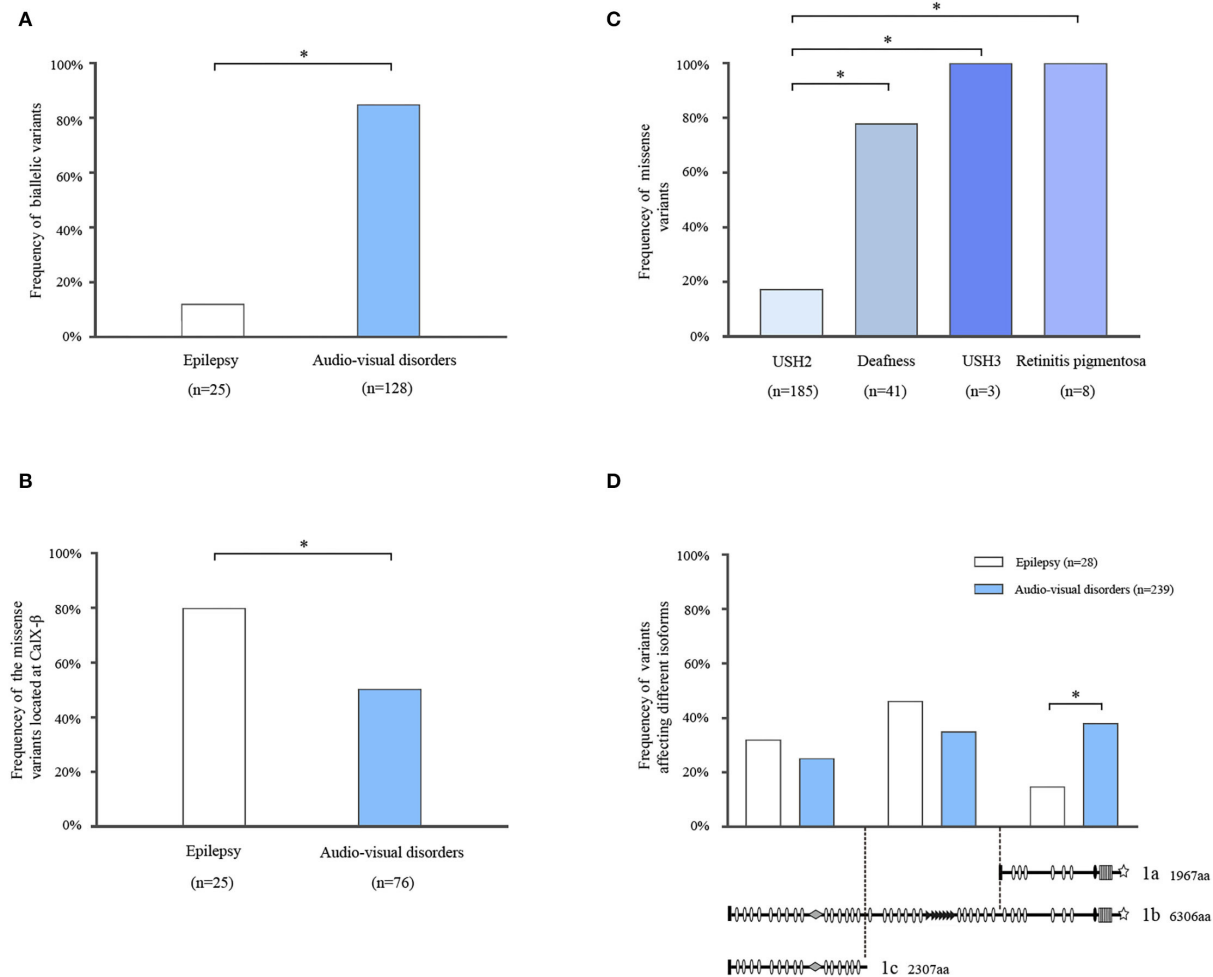
Systematic analysis of *ADGRV1* variants in epilepsy and audio-visual disorders. **(A)** The frequency of biallelic variants in epilepsy and audio-visual disorders. **(B)** The frequency of the missense variants located at the CalX-β motif in epilepsy and audio-visual disorders. **(C)** The frequency of missense variants in different phenotypes of audio-visual disorders. **(D)** The location distribution of the variants in epilepsy and audio-visual disorders. The values are expressed as the percentage of the variants located at the N-terminal segment (residues 1–2,295), central segment (residues 2,296–4,339), and C-terminal segment (residues 4,340–6,306). Fisher's exact test and chi-square test were used for statistical analysis. *means *P* < 0.05.

Among the cases with audio-visual abnormalities, USH2 was the most common phenotype with 98 cases reported. There were 23 cases with nonsyndromic hearing loss, four cases with nonsyndromic retinitis pigmentosa, and three cases with USH3. Additionally, two cases with *ADGRV1* variants were reported as unclassified USH, which were not included for further analysis due to the lack of clinic details. The majority of patients with audio-visual disorders (85.16%, 109/128) had biallelic variants (i.e., homozygous and compound heterozygous). In contrast, monoallelic variants were more common in patients with epilepsy (88.00%, 22/25) (Figure 4A).

CalX-β motifs are the most recurrent function domains in the ectodomain of VLGR1b. To explore the correlation between the dysfunction of CalX-β motifs and diseases, we analyzed the location of the missense variants identified in different phenotypes (Supplementary Table 3). Compared with audio-visual disorders (51.32%, 39/76), the frequency of missense variants located at the CalX-β motif was significantly higher in epilepsy (80.00%, 20/25) (Figure 4B).

To explore the relationship between genotype and phenotypic severity among audio-visual disorders, we analyzed the variant constituents of USH2, nonsyndromic deafness, nonsyndromic retinitis pigmentosa, and USH3. Missense variants accounted for 18.38% (34/185) of the variants in USH2, whereas it was 78.05% (32/41) in nonsyndromic hearing loss, and it occurred as the unique variant type in nonsyndromic retinitis pigmentosa (8/8) and USH3 (3/3). There was a significant difference in the frequencies of missense variants between USH2 and nonsyndromic hearing loss or nonsyndromic retinitis pigmentosa or USH3 (Figure 4C).

Three main mRNA isoforms, namely VLGR1a, VLGR1b, and VLGR1c, are expressed in humans. Variants at the N-terminal segment (residues 1–2,295) potentially affect isoforms VLGR1b and 1c. Variants at the central segment (residues 2,296–4,339) affect only isoform VLGR1b. Variants at the C-terminal segment (residues 4,340–6,306) affect isoforms VLGR1b and 1a (Figure 4D). The frequency of the variants located at the C-terminal segment was significantly higher in audio-visual disorders (38.91%, 93/239) than that in epilepsy (17.85%, 5/28) (Figure 4D). Subsequently, the variants associated with epilepsy mainly affected VLGR1b and 1c rather than VLGR1a.

## Discussion

This study identified *ADGRV1* variants in nine unrelated cases with FS-related epilepsy, including two heterozygous frameshift variants, six heterozygous missense variants, and a pair of compound heterozygous missense variants. The aggregate frequency of these variants in the case-cohort was significantly higher than that in control populations. The missense variants were located in the functional domains and were predicted to affect the molecular structures by changing the original hydrogen bonds. These clues suggested that *ADGRV1* variants were potentially associated with epilepsy. However, these variants were inherited from their asymptomatic parents, and the affected patients presented few seizures and responded well to AEDs. The incomplete penetrance and mild phenotype indicated that *ADGRV1* variants potentially caused changes in susceptibility.

Febrile seizures are the most common convulsive events in childhood, which may be accompanied by unprovoked seizures and epilepsy. Previously, five genetic loci have been reported to be responsible for FS including *FEB1* on chromosome 8q13–21, *FEB2* on chromosome 19p13.3, *FEB3* on chromosome 2q23–24, *FEB4* on chromosome 5q14-15, and *FEB5* on chromosome 6q22–24 (Johnson et al., 1998; Peiffer et al., 1999; Nakayama et al., 2000). Genes potentially associated with FS-related epilepsy include *SCN1A, ADGRV1, SCN1B, SCN9A, GABRG2, GABRD*, and *CPA6* (Wang et al., 2017). *SCN1A* variants are the most common causes of FS-related epilepsy, with more than 1,200 variants identified (www.gzneurosci.com/scn1a/database/) (Meng et al., 2015). In this cohort, 24.75% of patients had *SCN1A* variants, which confirmed the causative role of *SCN1A* in FS-related epilepsy. *ADGRV1* variants were identified in 8.91% of the cases and listed as the second, suggesting that *ADGRV1* was one of the candidate genes associated with FS or FS-related epilepsy. The incomplete penetrance suggests that *ADGRV1* variants caused a relatively lower pathogenicity (susceptibility) to epilepsy, coincident with the relatively mild phenotype of FS-related epilepsy shown in this study.

The patients with *ADGRV1* variants presented favorable responses to AEDs, including sodium channel blocker AEDs. In contrast, most patients with FS and FS-related epilepsy caused by *SCN1A* variants, such as Dravet syndrome (Brunklaus et al., 2012) and partial epilepsy with FS plus (Liao et al., 2010), were at risk of seizure aggravation induced by sodium channel blocker AEDs. Therefore, the present findings implied the significance of genetic testing in clinical treatment and management.

*ADGRV1* variants p.Asn2521IlefsX19 and p.Ile3575MetfsX2 resulted in the massive deletion of the main functional domains of the VLGR1 protein. Similarly, a nonsense variant p.S2832X of *ADGRV1* (p.S2652X in the *MASS1* isoform) was identified in two FS-affected siblings (Nakayama et al., 2002). Taken together with the evidence from genetic experiments that *Mass1* truncating mutation caused audiogenic seizures in the Frings mouse (Skradski et al., 2001; McMillan and White, 2004; Yagi et al., 2009), it is suggested that the loss of function or haploinsufficiency of *ADGRV1* potentially contributed to the epileptogenesis. Except for p.Leu15Ile and p.Ala3234Val, the remaining twelve missense variants, including six possible pathogenic variants reported previously (Myers et al., 2018), were located at or close to CalX-β motifs and proposed to affect the structures. This evidence suggested that CalX-β motifs were critical for VLGR1 function, and missense variants ruining the CalX-β motif were potentially associated with epilepsy. Indeed, the maintenance and the existence of a highly repeated structure of the VLGR1 protein, such as the multiple CalX-β motifs, were suggested to be essential for protein function (McMillan et al., 2002). The variant p.Ala3234Val was located at epilepsy-associated repeat 1, which is a common domain that existed in proteins encoded by epilepsy-associated genes such as *LGI1* and was proposed to play an important role in the pathogenesis of epilepsy (Staub et al., 2002). Further functional studies are required to determine the impacts of the variants on these functional domains and their roles in epileptogenesis.

On the contrary, most of the currently reported *ADGRV1* variants have been identified in audio-visual disorders. Further analysis demonstrated that biallelic variants were more common in audio-visual disorders than epilepsy (Figure 4A). For audio-visual disorders, a destructive variant was the major genotype of the severe phenotype (USH2). In contrast, missense variants were identified in most cases with relatively mild phenotypes, including nonsyndromic hearing loss, nonsyndromic retinitis pigmentosa, and USH3 (Figure 4C). These findings suggest that the genetic impairment of *ADGRV1* was associated with the phenotypic severity of audio-visual disorders, particularly concerning hearing loss.

The patients with FS-related epilepsy in this study did not appear any obvious audio-visual symptoms. Subclinical auditory and visual abnormalities were observed in further tests. Mild hearing impairment was detected in the patient with the heterozygous frameshift variant. Both mild hearing impairment and moderate retinitis pigmentosa were detected in the patient with compound heterozygous variants. Among the patients with the heterozygous missense variant, the patient with the hydrogen bond-changed variant presented horizontal semicircular canal weakness, while the patients carrying the variant without a hydrogen bond change did not suffer from any auditory or visual abnormality. These observations were consistent with the genotype-phenotype correlation between *ADGRV1* and audio-visual disorders. There was the possibility that the hearing impairment and retinitis pigmentosa would aggregate later, like the patients with USH2 or USH3, and the auditory and visual abnormalities might influence the learning and social abilities. It is, therefore, recommended to follow up the patients with *ADGRV1* variants with auditory and visual tests.

A correlation between the severity of the epilepsy phenotype and *ADGRV1* impairment was also suggested in this study. Cases with heterozygous variants presented relatively mild seizures and a good response to AEDs than the patient with compound heterozygous variants, who experienced relatively refractory seizures, and seizure-free was achieved after the combination treatment of AEDs. Additionally, cases 4 and 7 presented normal EEGs and became seizure-free without any treatment, in whom the variants (i.e., p.Thr2690Ala and p.Ala3234Val) did not change the hydrogen bonds in protein modeling. However, the severe phenotype of epilepsy had not been observed in the USH2 cases that carried *ADGRV1* variants of severe genetic impairment. The mechanism underlying the perplexing phenomenon is unknown, for which two clues from this study may be helpful to explain.

First, the isoforms involved may differ in audio-visual disorders and epilepsy. This study showed that the variants in isoforms VLGR1b and VLGR1c rather than that in VLGR1a appeared more frequently in patients with epilepsy (Figure 4D). A previous study suggested that *ADGRV1* variants in USH2 involved isoforms VLGR1b and 1a (Weston et al., 2004). The tissue-specific expression of isoforms is potentially the pathogenic bases of the diverse phenotypic spectrum associated with *ADGRV1*. The RT-PCR study on the mouse embryo demonstrated that Vlgr1b and Vlgr1c were expressed predominantly in the brain ventricular zone and participated in the neurogenesis process (McMillan et al., 2002). Both *Vlgr1*-knockout and recombinant mutant mice presented high susceptibility to audiogenic seizures. This experimental evidence suggested that the isoforms VLGR1b and VLGR1c were associated with the pathogenesis of epilepsy. In contrast, several experimental studies demonstrated that VLGR1a and VLGR1b were critical for the pathogenesis of audio-visual disorders. In hair cells of cochlea, VLGR1 mainly localizes at the ankle region of the stereocilia (McGee et al., 2006). The PDZ domain-binding motifs at the C-terminal end of VLGR1a and VLGR1b have been identified to mediate the interaction with several proteins, the majority of which are members of the ankle-link complex in stereocilia of hair cells. The ankle-link complex plays crucial roles in maintaining the stereociliary integrity and stability and in the hearing signal transduction process (Goodyear et al., 2005). Therefore, the structures and function of VLGR1a and VLGR1b supported their roles in hair cells and auditory disorders.

Second, this study showed that the epilepsy-associated missense variants occurred more frequently in the CalX-β motif than that in audio-visual disorders (Figure 4B). The extracellular domain of VLGR1b contains 35 CalX-β motifs, which resemble the regulatory domains of Na^+^/Ca^2+^ exchangers (Nikkila et al., 2000). In the central nervous system, Na^+^-Ca^2+^ exchanges play a fundamental role in controlling changes in the intracellular concentrations of Na^+^ and Ca^2+^ ions that occur in physiologic conditions such as neurotransmitter release, cell migration and differentiation, and gene expression, as well as neurodegenerative processes (Canitano et al., 2002). Therefore, the disrupted function caused by variants in the CalX-β motif was potentially involved with epileptogenesis.

This study has several limitations. Functional studies are needed to determine the damage effects of the variants. The relationships between the functional domains of VLGR1 and epilepsy also warrant further studies. The audio-visual abnormalities in patients with *ADGRV1* variants should be followed up.

In conclusion, we identified 10 *ADGRV1* variants in nine unrelated cases with FS or epilepsy with antecedent FS. The incomplete penetrance and mild phenotype indicated that *ADGRV1* variants potentially caused changes in susceptibility. The genotype, submolecular implication, isoforms, and damaging severity of the variants explained the phenotypical variations. *ADGRV1* variants associated with FS/epilepsy presented favorable responses to AEDs, implying a clinical significance.

## Data Availability Statement

The datasets presented in this study can be found in online repositories. The names of the repository/repositories and accession number(s) can be found at: https://www.ncbi.nlm.nih.gov/nuccore/, ON156994-ON157024.

## Ethics Statement

The studies involving human participants were reviewed and approved by the Ethics Committee of the Second Affiliated Hospital of Guangzhou Medical University and Guangdong Provincial People's Hospital. Written informed consent to participate in this study was provided by the participants' legal guardian/next of kin. Written informed consent was obtained from the individual(s), and minor(s)' legal guardian/next of kin, for the publication of any potentially identifiable images or data included in this article.

## Author Contributions

The study was conceived by YY. The case collection was carried out by BL, PZ, HM, LY, JZ, QZ, YY, NH, XS, WZ, and BH. Variant screening and data analysis were performed by PZ, HM, XLi, WB, ZL, XLe, BT, and TS. The manuscript was written by PZ, HM, and YY. Protein structure modeling was carried out by HL and YM. All authors contributed to the article and approved the submitted version.

## Funding

This study was supported by grants from the National Natural Science Foundation of China (Grant Nos. 81870903, 81971216, and 82071548), National Key Research and Development Program of China (Grant No. 2016YFC1306200), Natural Science Foundation of Guangdong Province (Grant No. 2020A1515010108), Science and Technology Project of Guangzhou (Grant Nos. 201904010292 and 201904020028), Science and Technology Project of Guangdong Province (Grant No. 2017B030314159), and Multi-Center Clinical Research Fund Project of the Second Affiliated Hospital of Guangzhou Medical University (Grant No. 2020-LCYJ-DZX-03). The funders had no role in the study design, data collection and analysis, and decision to publish or preparation of the manuscript.

## Conflict of Interest

HL and YM were employed by BGI-Shenzhen. The remaining authors declare that the research was conducted in the absence of any commercial or financial relationships that could be construed as a potential conflict of interest.

## Publisher's Note

All claims expressed in this article are solely those of the authors and do not necessarily represent those of their affiliated organizations, or those of the publisher, the editors and the reviewers. Any product that may be evaluated in this article, or claim that may be made by its manufacturer, is not guaranteed or endorsed by the publisher.
