# Supplementary material for: ADGRV1 Variants in Febrile Seizures/Epilepsy With Antecedent Febrile Seizures and Their Associations With Audio-Visual Abnormalities
Source: Front Mol Neurosci. 2022 Jun 23;15:864074. doi: 10.3389/fnmol.2022.864074 (PMC9262510; doi:10.3389/fnmol.2022.864074)
Supplement: Supplementary file 1 [file Table_1.DOCX]

**Supplementary Table 1.** **List of the 480 genes included on the present panel.**

| ABAT | ATP1A3 | CLEC18A | EHMT1 | GBA | HEXB | KCNJ2 | MAP2 |
| --- | --- | --- | --- | --- | --- | --- | --- |
| ABCC2 | ATP2A2 | CLIC2 | EIF2AK3 | GCDH | HLCS | KCNJ3 | MBD5 |
| ABCC8 | ATP6AP2 | CLN3 | ELOVL4 | GCH1 | HNRNPU | KCNJ5 | MBTPS2 |
| ACADM | ATP6V0A2 | CLN5 | ELP4 | GCM2 | HOXA1 | KCNJ6 | ME2 |
| ACO2 | ATP7A | CLN6 | EMX2 | GCSH | HPD | KCNJ9 | MECP2 |
| ACOX1 | AUH | CLN8 | EN2 | GJA1 | HSD17B10 | KCNK1 | MED17 |
| ACP1 | BCKDK | CNP | EPM2A | GJD2 | HTR1A | KCNK3 | MEF2C |
| ACSF3 | BDNF | CNR1 | EPM2AIP1 | GLB1 | IDH2 | KCNK7 | MFSD8 |
| ACTA2 | BRAT1 | CNTF | FADD | GLDC | IDS | KCNK9 | MIB1 |
| ACTB | BRD2 | CNTN2 | FARS2 | GLRA1 | IER3IP1 | KCNMA1 | MMADHC |
| ACY1 | BTD | CNTNAP2 | FKTN | GLRB | IL6ST | KCNMB2 | MOCS1 |
| ADAR | C10ORF2 | COG6 | FLNA | GLUD1 | IMPA2 | KCNMB3 | MOCS2 |
| ADCK3 | CA1 | COG8 | FOLR1 | GM2A | INA | KCNMB4 | MOG |
| ADGRV1 | CACNA1A | COL4A1 | FOS | GOSR2 | IQSEC2 | KCNN2 | MSN |
| ADK | CACNA1E | COQ2 | FOXG1 | GPHN | JRK | KCNQ1 | MTHFR |
| ADSL | CACNA1G | COQ9 | FUCA1 | GPR56 | JUN | KCNQ2 | MTMR9 |
| AFG3L2 | CACNA1H | CPA6 | GABBR1 | GRIA1 | KARS | KCNQ3 | MTR |
| AGTR2 | CACNA2D2 | CPS1 | GABBR2 | GRIA2 | KCNA1 | KCNQ4 | NAGA |
| ALDH4A1 | CACNG2 | CPT2 | GABRA1 | GRIA3 | KCNA2 | KCNQ5 | NDE1 |
| ALDH5A1 | CALHM1 | CSTB | GABRA2 | GRIA4 | KCNA6 | KCNS2 | NDP |
| ALDH7A1 | CASK | CTDP1 | GABRA3 | GRIK1 | KCNAB1 | KCNS3 | NDUFV1 |
| ALG13 | CASP9 | CTSD | GABRA4 | GRIK2 | KCNAB2 | KCNT1 | NF1 |
| AMACR | CASR | CUL4B | GABRA5 | GRIN1 | KCNAB3 | KCNV1 | NGLY1 |
| AMT | CBS | CYP4F11 | GABRA6 | GRIN2A | KCNB1 | KCNV2 | NHLRC1 |
| ANK3 | CCDC88C | D2HGDH | GABRB1 | GRIN2B | KCNC1 | KCTD7 | NIPA2 |
| ANKRD11 | CCL3 | DCX | GABRB2 | GRIN2C | KCNC2 | KDM5C | NPY |
| AP4E1 | CCL4 | DDC | GABRB3 | GRIN2D | KCNC4 | KLK1 | NRXN1 |
| ARG1 | CCM2 | DEPDC5 | GABRD | GRIN3A | KCND1 | KRIT1 | NSDHL |
| ARHGEF15 | CDK5 | DGKD | GABRE | GRIN3B | KCND2 | L1CAM | NSF |
| ARHGEF9 | CDKL5 | DHFR | GABRG1 | GRM1 | KCNE1L | LAMA2 | NTRK1 |
| ARSA | CHD2 | DIAPH3 | GABRG2 | GRN | KCNE2 | LAMB1 | NTRK2 |
| ARX | CHRFAM7A | DLX2 | GABRG3 | HAX1 | KCNF1 | LBR | OPA1 |
| ASAH1 | CHRNA2 | DLX5 | GABRP | HCCS | KCNG1 | LGI1 | OPHN1 |
| ASIC1 | CHRNA4 | DNM1 | GABRQ | HCFC1 | KCNG4 | LGI2 | OPRM1 |
| ASPA | CHRNA5 | DPM1 | GABRR1 | HCN1 | KCNH2 | LGI4 | OTX2 |
| ASPM | CHRNA7 | DPYS | GABRR2 | HCN2 | KCNH3 | LIAS | PAFAH1B1 |
| ASS1 | CHRNB2 | DYRK1A | GABRR3 | HCN4 | KCNH8 | LIFR | PAH |
| ATIC | CLCN2 | EFHC1 | GAD1 | HDAC4 | KCNJ1 | LMBRD1 | PALLD |
| ATN1 | CLCN4 | EFHC2 | GAMT | HEPACAM | KCNJ10 | MANBA | PARK2 |
| ATP1A2 | CLCNKB | EGF | GATM | HEXA | KCNJ11 | MAOB | PC |

| PCDH19 | PHGDH | PRNP | ROGDI | SLC12A5 | SLC6A13 | SV2A | TSC1 |
| --- | --- | --- | --- | --- | --- | --- | --- |
| PDCD10 | PHOX2A | PRODH | RPS6KA3 | SLC12A6 | SLC6A19 | SYN1 | TSC2 |
| PDHA1 | PIGA | PRRT2 | SCARB2 | SLC16A1 | SLC6A8 | SYNGAP1 | TSEN2 |
| PDHX | PIGL | PSAP | SCN1A | SLC17A5 | SLC9A3 | SYP | TSEN34 |
| PDYN | PIGN | PSAT1 | SCN1B | SLC19A3 | SLC9A6 | SYT11 | TSEN54 |
| PEX1 | PIGO | PSEN1 | SCN2A | SLC1A1 | SLC9A9 | SZT2 | TSPO |
| PEX10 | PIGV | PTEN | SCN2B | SLC1A2 | SMARCA2 | TBC1D24 | TUBA1A |
| PEX12 | PIR | PTH | SCN3A | SLC1A3 | SMARCA4 | TBCE | TUBA8 |
| PEX13 | PKHD1 | PTS | SCN3B | SLC20A2 | SNIP1 | TBX1 | TUBB2B |
| PEX14 | PLCB1 | QDPR | SCN4A | SLC25A12 | SOBP | TCF4 | TUBGCP6 |
| PEX16 | PNKD | RAB39B | SCN5A | SLC25A13 | SPAST | TICAM1 | U2AF1 |
| PEX19 | PNKP | RANBP2 | SCN7A | SLC25A22 | SPR | TK2 | UBC |
| PEX2 | PNPO | RBFOX1 | SCN8A | SLC2A1 | SPTAN1 | TLR3 | UBE2A |
| PEX26 | POLG | RBPJ | SCN9A | SLC35A2 | SRGAP2 | TMEM67 | UBE3A |
| PEX3 | POLG2 | RELN | SDHA | SLC35A3 | SRPX2 | TNF | VAMP2 |
| PEX5 | PPP1R3C | RHAG | SEPSECS | SLC35C1 | ST3GAL3 | TPP1 | VLDLR |
| PEX6 | PPT1 | RHOA | SERPINI1 | SLC46A1 | ST3GAL5 | TPRXL | VPS13A |
| PFKL | PRF1 | RMND1 | SEZ6 | SLC4A3 | STRADA | TREX1 | WARS |
| PFKM | PRICKLE1 | RNASEH2A | SHANK3 | SLC52A2 | STXBP1 | TRMT44 | WDR45 |
| PHF6 | PRICKLE2 | RNF213 | SLC12A1 | SLC6A11 | SUOX | TRPM6 | ZEB2 |
