# Supplementary material for: ADGRV1 Variants in Febrile Seizures/Epilepsy With Antecedent Febrile Seizures and Their Associations With Audio-Visual Abnormalities
Source: Front Mol Neurosci. 2022 Jun 23;15:864074. doi: 10.3389/fnmol.2022.864074 (PMC9262510; doi:10.3389/fnmol.2022.864074)
Supplement: Supplementary file 2 [file Table_2.DOCX]

**Supplementary table 2. The variants of *SCN1A* and *SCN9A* identified in this study.**

| **Case** | **Diagnosis** | **Gene** | **Variant** | **Inheritance** | **MAF** | **SIFT** | **PP2_Var** | **Mutation Taster** | **CADD** | **Fathhmm-MKL** | **GERP++** | **PhyloP** | **PhastCons** |
| --- | --- | --- | --- | --- | --- | --- | --- | --- | --- | --- | --- | --- | --- |
| 1 | **FS** | *SCN1A* | c.4310T>C/p.Ile1437Thr | Paternal | **-** | D | D | D | D | D | C | C | C |
| 2 | **FS** | *SCN1A* | c.2947-3A>G | *De novo* | **-** | - | - | - | - | - | - | - | - |
| 3 | **FS+** | *SCN1A* | c.2035C>G/p.Pro679Ala | Maternal | **-** | D | D | D | D | D | C | C | C |
| 4 | **FS+** | *SCN1A* | c.3361G>T/p.Glu1121X | *De novo* | **-** | - | - | D | D | D | C | C | C |
| 5 | **FS+** | *SCN1A* | c.2345C>G/p.Thr782Ser | Maternal | **-** | D | D | D | D | D | C | C | C |
| 6 | **FS+** | *SCN1A* | c.1658delA/p.His553ProfsX5 | *De novo* | **-** | - | - | D | - | - | - | **-** | **-** |
| 7 | **FS+** | *SCN1A* | c.4476+1A>G | *De novo* | **-** | - | - | - | - | - | - | **-** | **-** |
| 8 | **FS+** | *SCN1A* | c.3985C>T/p.Arg1329X | *De novo* | **-** | - | - | D | D | D | C | C | C |
| 9 | **FS+** | *SCN1A* | c.3767A>C/p.Asp1256Ala | *De novo* | **-** | D | D | D | D | D | C | C | C |
| 10 | **FS+** | *SCN1A* | c.2827_2830del/p.Ile943CysfsX10 | *De novo* | **-** | - | - | D | - | - | - | **-** | **-** |
| 11 | **FS+** | *SCN1A* | c.823A>G/p.Asn275Asp | *De novo* | **-** | D | B | D | D | D | C | NC | C |
| 12 | **FS+** | *SCN1A* | c.1130G>C/p.Arg377Pro | *De novo* | **-** | D | D | D | D | D | C | C | C |
| 13 | **FS+** | *SCN1A* | c.1624C>T/p. Arg542X | *De novo* | **-** | - | - | D | D | D | NC | C | C |
| 14 | **FS+** | *SCN1A* | c.3862A>G/p.Arg1288Gly | *De novo* | **-** | D | D | D | D | D | C | C | C |
| 15 | **FS+** | *SCN1A* | c.3905A>T/p.Lys1302Ile | *De novo* | **-** | D | D | D | D | D | C | C | C |
| 16 | **FS+** | *SCN1A* | c.4061G>T/p.Gly1354Val | *De novo* | **-** | D | D | D | D | D | C | C | C |
| 17 | **FS+** | *SCN1A* | c.4219C>T/p.Arg1407X | Maternal* | **-** | - | - | D | D | D | C | C | C |
| 18 | **FS+** | *SCN1A* | c.4282G>T/p.Vla1428Phe | *De novo* | **-** | D | D | D | D | D | C | C | C |
| 19 | **FS+** | *SCN1A* | c.5029C>T/p.Leu1677Phe | *De novo* | **-** | D | D | D | D | D | C | C | C |
| 20 | **FS+** | *SCN1A* | c.3631G>A/p.Asp1211Asn | *De novo* | **-** | D | D | D | D | D | C | C | C |
| 21 | **FS+** | *SCN1A* | c.302G>A/p.Arg101Gln | *De novo* | **-** | D | D | D | D | D | C | C | C |
| 22 | **FS+** | *SCN1A* | c.4136_4140del/p.Thr1379fsX | *De novo* | - | - | - | D | - | - | - | - | - |
| 23 | **FS+** | *SCN1A* | c.5156A>C/p.Gln1719Pro | Maternal* | **-** | D | B | D | D | D | C | C | C |
| 24 | **FS+** | *SCN1A* | c.1852C>T/p.Arg618Cys | Paternal | **-** | D | D | D | D | D | C | C | C |
| 25 | **FS+** | *SCN1A* | c.4391T>C/p.Val1464Ala | Maternal | **-** | D | D | D | D | D | C | C | C |
| 26 | **FS** | *SCN9A* | c.2198C>G/p.Pro733Arg | Maternal | **-** | D | D | D | D | D | C | C | C |
| 27 | **FS+** | *SCN9A* | c.77G>A/p.Arg26His | Maternal | **-** | D | D | D | D | D | C | C | C |
| 28 | **FS+** | *SCN9A* | c.3712G>T/p.Gly1238Cys | Paternal | **-** | D | D | D | D | D | C | C | C |

Abbreviations: B, benign; C, conserved; CADD, Combined Annotation Dependent Depletion; D, damaging; MAF, minor allele frequency from gnomAD; NC, non-conserved; PP2_Var, Polyphen2_HVAR.

*affected
