# Supplementary material for: ADGRV1 Variants in Febrile Seizures/Epilepsy With Antecedent Febrile Seizures and Their Associations With Audio-Visual Abnormalities
Source: Front Mol Neurosci. 2022 Jun 23;15:864074. doi: 10.3389/fnmol.2022.864074 (PMC9262510; doi:10.3389/fnmol.2022.864074)
Supplement: Supplementary file 3 [file Table_3.docx]

**Supplementary Table 3. *ADGRV1* variants in audio-visual disorders and epilepsy**

| **Phenotype** | **Variant** | **Genotype** | **Zygosity** | **Location** | **Affected isoform(s)** | **Ref.** |
| --- | --- | --- | --- | --- | --- | --- |
| **Audio-visual disorder** | | | | | |  |
| **USH2** | | | | | |  |
| 1 | c.4400G>A(p.Gly1476Asp) | missense | homozygous | Calx-*β*10 | 1b,1c | (Gloeckle et al., 2014) |
| 2 | c.10019T>G(p.Vla3340Gly) | missense | homozygous | EAR2 | 1b | (Kahrizi et al., 2014) |
| 3 | c.14159C>T(p.Pro4720Leu) | missense | homozygous | Calx-*β*32 | 1b,1a | (Fuster-Garcia et al., 2018) |
| 4 | c.14366G>A(p.Arg4789Gln) | missense | homozygous | Calx-*β*32～Calx-*β*33 | 1b,1a | (Gu et al., 2015) |
| 5 | c.18131A>G(p.Tyr6044Cys) | missense | homozygous | TM5 | 1b,1a | (Hmani-Aifa et al., 2009) |
| 6 | c.1608C>G(p.Tyr536X) | nonsense | homozygous | - | 1b,1c | (Bonnet et al., 2016) |
| 7 | c.2864C>A(p.Ser955X) | nonsense | homozygous | - | 1b,1c | (Besnard et al., 2014; Bonnet et al., 2016) |
| 8 | c.6901C>T(p.Gln2301X) | nonsense | homozygous | - | 1b | (Weston et al., 2004) |
| 9 | c.7606G>T(p.Glu2536X) | nonsense | homozygous | - | 1b | (Neuhaus et al., 2017) |
| 10 | c.10468C>T(p.Gln3490X) | nonsense | homozygous | - | 1b | (Bonnet et al., 2016) |
| 11 | c.14971C>T(p.Arg4991X) | nonsense | homozygous | - | 1b,1a | (Sun et al., 2018) |
| 12 | c.16876C>T(p.Gln5626X) | nonsense | homozygous | - | 1b,1a | (Bonnet et al., 2016) |
| 13 | c.18054G>A(p.Trp6018X) | nonsense | homozygous | - | 1b,1a | (Fuster-Garcia et al., 2018) |
| 14 | c.2984_2988delGAGG(p.Leu995HisfsX2) | frameshift | homozygous | - | 1b,1c | (Besnard et al., 2012) |
| 15 | c.6912dupG (p.Leu2305ValfsX4) | frameshift | homozygous | - | 1b | (Wei et al., 2018) |
| 16 | c.8992_8994indel(p.Ala2998fs) | frameshift | homozygous | - | 1b | (Besnard et al., 2014) |
| 17 | c.13536_13537delTC(p.Pro4513LeufsX16) | frameshift | homozygous | - | 1b,1a | (Besnard et al., 2014) |
| 18 | c.15448_15449delCT(p.Leu5150HisfsX6) | frameshift | homozygous | - | 1b,1a | (Santana et al., 2019) |
| 19 | c.16040delA(p.Asp5347ValfsX22) | frameshift | homozygous | - | 1b,1a | (Reddy et al., 2014) |
| 20 | c.17368_17369delinsTTAT(p.Ser5790LeufsX6) | frameshift | homozygous | - | 1b,1a | (Garcia-Garcia et al., 2013) |
| 21 | c.17668_17669delAT(p.Met5890ValfsX10) | frameshift | homozygous | - | 1b,1a | (Bonnet et al., 2016) |
| 22 | c.18261delA(p.Asn6088SerfsX20) | frameshift | homozygous | - | 1b,1a | (Garcia-Garcia et al., 2013) |
| 23 | c.6932_6939dup(p.Glu2314ThrfsX13) | frameshift | homozygous | - | 1b | (Garcia-Garcia et al., 2013) |
| 24 | c.17756-2239_17856+11702delins17bp (p.Gly5919AsnfsX15) | frameshift | homozygous | - | 1b,1a | (Reddy et al., 2014) |
| 25 | c.12528-1G>T(p.?) | splice | homozygous | - | 1b | (Garcia-Garcia et al., 2013) |
| 26 | c.17204+4_17204+7del(p.?) | splice | homozygous | - | 1b,1a | (Garcia-Garcia et al., 2013) |
| 27 | c.12226_12228delATAins1 8(p.Ile4076delinsVDESRX) | indel | homozygous | - | 1b | (Gloeckle et al., 2014) |
| 28 | Exon28_33del | gross deletion | homozygous | - | 1b | (Fuster-Garcia et al., 2018) |
| 29 | Exon83del | gross deletion | homozygous | - | 1b,1a | (Stabej et al., 2012) |
| 30 | Exons84_85del | gross deletion | homozygous | - | 1b,1a | (Hilgert et al., 2009) |
| 31 | Exons79_83dup | gross duplication | homozygous | - | 1b,1a | (Aparisi et al., 2014) |
| 32 | c.6307G>T(p.Glu2103X)  +c.6901C>T(p.Gln2301X) | nonsense  nonsense | compound heterozygous | -  - | 1b,1c  1b | (Stabej et al., 2012) |
| 33 | c.17488C>T(p.Gln5830X)  +c.18310G>T(p.Glu6104X) | nonsense  nonsense | compound heterozygous | -  - | 1b,1a  1b,1a | (Krawitz et al., 2014) |
| 34 | c.929G>A(p.Gly310Glu)  +c.13048T>C(p.Ser4350Pro) | missense  missense | compound heterozygous | Calx-*β*3  Calx-*β*29 | 1b,1c  1b,1a | (Jiang et al., 2015) |
| 35 | c.2277T>A( p.Tyr759X)  + c.17933A>G(p.His5978Arg) | nonsense  missense | compound heterozygous | -  E | 1b,1c  1b,1a | (Sloan-Heggen et al., 2016) |
| 36 | c.2398C>T(p.Arg800X)  +c.13433G>T(p.Ser4478Ile) | nonsense  missense | compound heterozygous | -  Calx-*β*30 | 1b,1c  1b,1a | (Stabej et al., 2012) |
| 37 | c.5330T>G(p.Leu1777X)  +c.4378G>A(p.Gly1460Ser) | nonsense  missense | compound heterozygous | -  Calx-*β*10 | 1b,1c  1b,1c | (Magliulo et al., 2017) |
| 38 | c.6901C>T(p.Gln2301X)  + c.746G>A(p.Arg249Lys) | nonsense  missense | compound heterozygous | -  Calx-*β*2～Calx-*β*3 | 1b  1b,1c | (Turro et al., 2020) |
| 39 | c.6856C>T(p.Arg2286X)  +c.10016G>A(p.Ser3339Asn) | nonsense  missense | compound heterozygous | -  EAR2 | 1b  1b | (Stabej et al., 2012) |
| 40 | c.7001T>G(p.Leu2334X)  +c.17933A>G(p.His5978Arg) | nonsense  missense | compound heterozygous | -  E | 1b  1b,1a | (Besnard et al., 2012) |
| 41 | c.9877C>T(p.Arg3293X)  +c.4072T>C(p.Ser1358Pro) | nonsense  missense | compound heterozygous | -  Calx-*β*9～Calx-*β*10 | 1b  1b,1c | (Bonnet et al., 2016) |
| 42 | c.12802C>T(p.Arg4268X)  +c.3917G>A(p.Gly1306Glu) | nonsense  missense | compound heterozygous | -  Calx-*β*9～Calx-*β*10 | 1b  1b,1c | (Lenarduzzi et al., 2019) |
| 43 | c.14404C>T(p.Arg4802X)  +c.9974T>C(p.Ile3325Thr) | nonsense  missense | compound heterozygous | -  EAR1 | 1b,1a  1b | (Stabej et al., 2012) |
| 44 | c.12143C>A(p.Ser4048X)  +c.11947G>A(p.Asp3992Asn) | nonsense  missense | compound heterozygous | -  Calx-*β*26 | 1b  1b | (Bonnet et al., 2016) |
| 45 | c.16886G>A (p.Trp5629X)  +c.4102A>T(p.Asn1368Tyr) | nonsense  missense | compound heterozygous | -  Calx-*β*9～Calx-*β*10 | 1b,1a  1b,1c | (Fuster-Garcia et al., 2018) |
| 46 | c.333_334delTT(p.Phe112fsX29)  +c.10196A>C(p.His3399Pro) | frameshift  missense | compound heterozygous | -  EAR4 | 1b,1c  1b | (Bonnet et al., 2011) |
| 47 | c.956dupA(p.Asn319LysfsX6)  + c.14365C>T(p.Arg4789Trp) | frameshift  missense | compound heterozygous | -  Calx-*β*32～Calx-*β*33 | 1b,1c  1b,1a | (Sloan-Heggen et al., 2016) |
| 48 | c.1701delC(p.Leu568CysfsX8)  +c.17909G>C(p.Cys5970Ser) | frameshift  missense | compound heterozygous | -  E | 1b,1c  1b,1a | (Bonnet et al., 2016) |
| 49 | c.3945dupA(p.Gln1316fs)  +c.17933A>G(p.His5978Arg) | frameshift  missense | compound heterozygous | -  E | 1b,1c  1b,1a | (Besnard et al., 2012) |
| 50 | c.10935_10938del(p.Ser3646MetfsX27)  +c.9042G>C(p.Met3014Ile) | frameshift  missense | compound heterozygous | -  Calx-*β*21 | 1b  1b | (Besnard et al., 2012) |
| 51 | c.16604_16611delGTACCCAG  (p.Ser5535ArgfsX6)  +c.9464C>A(p.Ala3155Asp) | frameshift  missense | compound heterozygous | -  Calx-*β*22 | 1b,1a  1b | (Moteki et al., 2015) |
| 52 | c.1472delC(p.Thr491LysfsX8)  +c.16225C>T(p.Arg5409X) | frameshift  nonsense | compound heterozygous | -  - | 1b,1c  1b,1a | (Sun et al., 2018) |
| 53 | c.5624delG(p.Gly1875GlufsX13)  +c.7006C>T(p.Arg2336X) | frameshift  nonsense | compound heterozygous | -  - | 1b,1c  1b | (Sloan-Heggen et al., 2016) |
| 54 | c.8713_8716dup(p.Ile2906LysfsX6)  + c.6901C>T(p.Gln2301X) | frameshift  nonsense | compound heterozygous | -  - | 1b  1b | (Zampaglione et al., 2020) |
| 55 | c.13536_13537delTC(p.Pro4513LeufsX16)  +c.7129C>T(p.Arg2377X) | frameshift  nonsense | compound heterozygous | -  - | 1b  1b | (Besnard et al., 2014) |
| 56 | c.14451_14452del(p.Arg4819AlafsX11)  +c.7006C>T(p.Arg2336X) | frameshift  nonsense | compound heterozygous | -  - | 1b,1a  1b | (Jiang et al., 2015) |
| 57 | c.17668_17669del(p.Met5890ValfsX10)  +c.9877C>T(p.Arg3293X) | frameshift  nonsense | compound heterozygous | -  - | 1b,1a  1b | (Zampaglione et al., 2020) |
| 58 | c.2258_2270del13(p.Gln753LeufsX8)  +c.12961G>T(p.Glu4321X) | frameshift  nonsense | compound heterozygous | -  - | 1b,1c  1b,1a | (Bonnet et al., 2011) |
| 59 | c.18884_18887delAGCT(p.Glu6295AlafsX31)  +c.10458G>A(p.Trp3486X) | frameshift  nonsense | compound heterozygous | -  - | 1b,1a  1b | (Bonnet et al., 2016) |
| 60 | c.8716-8717insAACA(p.Ile2906fs)  +c.6901C>T(p.Gln2301X) | frameshift  nonsense | compound heterozygous | -  - | 1b  1b | (Weston et al., 2004) |
| 61 | c.15981delC(p.Phe5328SerfsX41)  +c.8005delG(p.Glu2669LysfsX4) | frameshift  frameshift | compound heterozygous | -  - | 1b,1a  1b | (Bonnet et al., 2016) |
| 62 | c.2258_2270del13(p.Gln753LeufsX8)  +c.5356_5357delAA(p.Lys1786IlefsX8) | frameshift  frameshift | compound heterozygous | -  - | 1b,1c  1b,1c | (Ebermann et al., 2009; Bonnet et al., 2016) |
| 63 | c.2145_2149delGTTTT(p.Leu715ProfsX6)  +c.2612delG(p.Gly871GlufsX8) | frameshift  frameshift | compound heterozygous | -  - | 1b,1c  1b,1c | (Aparisi et al., 2014) |
| 64 | c.6017del(p.Gly2006AlafsX13)  +c.7188_7189ins14(p.Val2397LeufsX2) | frameshift  frameshift | compound heterozygous | -  - | 1b,1c  1b | (Ammar-Khodja et al., 2015) |
| 65 | c.6962_63delTG(p.Val2321AlafsX4)  +c.15144delC(p.Ser5048ArgfsX29) | frameshift  frameshift | compound heterozygous | -  - | 1b  1b,1a | (Stabej et al., 2012) |
| 66 | c.10085_10088del(p.Val3363GlufsX11)  +c.10736_10737delCC(p.Ala3579ValfsX6) | frameshift  frameshift | compound heterozygous | -  - | 1b  1b | (Stabej et al., 2012) |
| 67 | c.1563dupT(p.Pro522fsX8)  +c.17668_17669delAT(p.Met5890ValfsX10) | frameshift  frameshift | compound heterozygous | -  - | 1b,1c  1b,1a | (Bonnet et al., 2011) |
| 68 | c.10085_10088del(p.Val3363AspfsX11)  +c.18646delG(p.Ala6216HisfsX13) | frameshift  frameshift | compound heterozygous | -  - | 1b  1b,1a | (Ebermann et al., 2009) |
| 69 | c.12932delC(p.Ala4311GlufsX19)  +c.17668_17669delAT(p.Met5890ValfsX10) | frameshift  frameshift | compound heterozygous | -  - | 1b  1b,1a | (Baux et al., 2017) |
| 70 | c.13320dupC(p.Ser4441LeufsX9)  +c.16940delT(p.Val5647GlyfsX7) | frameshift  frameshift | compound heterozygous | -  - | 1b,1a  1b,1a | (Besnard et al., 2012; Bonnet et al., 2016) |
| 71 | c.14767del(p.Thr4923ProfsX8)  +c.17668_17669del(p.Met5890ValfsX10) | frameshift  frameshift | compound heterozygous | -  - | 1b,1a  1b,1a | (Bryant et al., 2018) |
| 72 | c.15008delG(p.Gly5003AlafsX13)  +c.18383_18386dupACAG(p.His6130GlnfsX84) | frameshift  frameshift | compound heterozygous | -  - | 1b,1a  1b,1a | (Zhang et al., 2018) |
| 73 | c.6981delT(p.Gly2328ValfsX7)  +c.14044-1G>A | frameshift  splice | compound heterozygous | -  - | 1b  1b,1a | (Neuhaus et al., 2017) |
| 74 | c.7770delC(p.Glu2591LysfsX18)  +c.17204+4_17204+7del(p.?) | frameshift  splice | compound heterozygous | -  - | 1b  1b,1a | (Besnard et al., 2012) |
| 75 | c.6093delA(p.Ala2032ArgfsX27)  +c.9184+3A>G(p.?) | frameshift  splice | compound heterozygous | -  - | 1b,1c  1b | (Bonnet et al., 2016) |
| 76 | c.6984_6985delTG(p.Gly2329ArgfsX17)  +c.9906+1G>A(p.?) | frameshift  splice | compound heterozygous | -  - | 1b  1b | (Bonnet et al., 2016) |
| 77 | c.15716delA(p.Asn5239ThrfsX19)  + c.17204+5G>C | frameshift  splice | compound heterozygous | -  - | 1b,1a  1b,1a | (Neuhaus et al., 2017) |
| 78 | c.5671A>T(p.Arg1891X)  +c.13232-3C>G(p.?) | nonsense  splice | compound heterozygous | -  - | 1b,1c  1b,1a | (Besnard et al., 2012) |
| 79 | c.6133G>T(p.Gly2045X)  +c.10054-1G>T(p.?) | nonsense  splice | compound heterozygous | -  - | 1b,1c  1b | (Bonnet et al., 2016) |
| 80 | c.1477C>T(p.Arg493X)  +c.1509+3A>G(p.?) | nonsense  splice | compound heterozygous | -  - | 1b,1c  1b,1c | (Bonnet et al., 2016) |
| 81 | c.8749G>T(p.Glu2917X)  + Del ex85 | nonsense  gross deletion | compound heterozygous | -  - | 1b  1b,1a | (Neuhaus et al., 2017) |
| 82 | c.17668_17669delAT(p.Met5890ValfsX10)  +c.17020-?_17856+?dup(p.Ile5674_Gln5952dup) | frameshift  gross duplication | compound heterozygous | -  - | 1b,1a  1b,1a | (Besnard et al., 2012) |
| 83 | c.1892delC(p.Pro631LeufsX62)  +Exon85del | frameshift  gross deletion | compound heterozygous | -  - | 1b,1c  1b,1a | (Fuster-Garcia et al., 2018) |
| 84 | c.12555_12556delGG(p.Glu4186GlyfsX17)  +c.(6951+1_6952-1)_(7133+1_7134-1)del(p.exon32del) | frameshift  gross deletion | compound heterozygous | -  - | 1b,1a  1b | (Bonnet et al., 2016) |
| 85 | c.10458G>A(p.Trp3486X)  +c.(3022+1_3023-1)_(16611+1_16612-1)dup | nonsense  gross duplication | compound heterozygous | -  - | 1b  1a,1b,1c | (Bonnet et al., 2016) |
| 86 | c.11547delA(P.Glu3849LysfsX27)  +c.7130G>A(p.Arg2377Gln) | frameshift  missense | compound heterozygous | -  Calx-*β*16～Calx-*β*17 | 1b  1b | (Jiang et al., 2015) |
| 87 | c.3635-2A>G(p.?)  +c.14365C>T(p.Arg4789Trp) | splice  missense | compound heterozygous | -  Calx-*β*32～Calx-*β*33 | 1b,1c  1b,1a | (Besnard et al., 2012) |
| 88 | c.3974C>T(p.Thr1325Met) | missense | heterozygous | Calx-*β*9～Calx-*β*10 | 1b,1c | (Krawitz et al., 2014) |
| 89 | c.11974G>A (p.Asp3992Asn) | missense | heterozygous | Calx-*β*26 | 1b | (Aparisi et al., 2014) |
| 90 | c.6901C>T(p.Gln2301X) | nonsense | heterozygous | - | 1b | (Weston et al., 2004) |
| 91 | c.11410C>T(p.Arg3804X) | nonsense | heterozygous | - | 1b | (Neuhaus et al., 2017) |
| 92 | c.12895C>T(p.Arg4299X) | nonsense | heterozygous | - | 1b | (Neuhaus et al., 2017) |
| 93 | c.12982G>T(p.Glu4328X) | nonsense | heterozygous | - | 1b | (Turro et al., 2020) |
| 94 | c. 17062C>T(p.Arg5688X) | nonsense | heterozygous | - | 1b,1a | (Besnard et al., 2012) |
| 95 | c.18732_18750del(p.Thr6244X) | nonsense | heterozygous | - | 1b,1a | (Weston et al., 2004) |
| 96 | c. 8790delC(p.Met2931TrpfsX11) | frameshift | heterozygous | - | 1b | (Weston et al., 2004; Malm et al., 2011) |
| 97 | c.12555_12556delGG(p.Glu4186GlyfsX17) | frameshift | heterozygous | - | 1b | (Bonnet et al., 2011) |
| 98 | c.9623+1G>A(p.?) | splice | heterozygous | - | 1b | (Stabej et al., 2012) |
|  |  |  |  |  |  |  |
| **USH3** | | | | | |  |
| 1 | c.3443G>A(p.Gly1148Asp) | missense | heterozygous | Calx-*β*9 | 1b,1c | (Aparisi et al., 2014) |
| 2 | c.5830G>A(p.Asp1944Asn) | missense | heterozygous | Calx-*β*13 | 1b,1c | (Bonnet et al., 2011) |
| 3 | c.14654A>G(p.Asn4885Ser) | missense | heterozygous | Calx-*β*32～Calx-*β*33 | 1b,1a | (Bonnet et al., 2011) |
|  |  |  |  |  |  |  |
| **Unclassified USH** | | | | | |  |
| 1 | c.10301delT(p.Leu3434TyrfsX34)  +c.12528-1G>T(p.?) | frameshift  splice | compound heterozygous | -  - | 1b  1b | (Garcia-Garcia et al., 2013) |
| 2 | c.17386C>T(p.Gln5796X) | nonsense | heterozygous | - | 1b,1a | (Garcia-Garcia et al., 2013) |
|  |  |  |  |  |  |  |
| **Deafness** | | | | | |  |
| 1 | c.6559A>G(p.Ile2187Val) | missense | homozygous | Calx-*β*15 | 1b,1c | (Yang et al., 2013) |
| 2 | c.1797A>T(p.Arg599Ser) | missense | homozygous | Calx-*β*4～Calx-*β*5 | 1b,1c | (Miyagawa et al., 2013) |
| 3 | c.8084T>G(p.Ile2695Ser) | missense | homozygous | Calx-*β*19 | 1b | (Baux et al., 2017) |
| 4 | c.961G>T(p.Asp321Tyr)  +c.9429A>T(p.Glu3143Asp) | missense  missense | compound heterozygous | Calx-*β*3  Calx-*β*22 | 1b,1c  1b | (Chen et al., 2013) |
| 5 | c.1522A>C(p.Ile508Leu)  + c.16331C>A(p.Thr5444Lys) | missense  missense | compound heterozygous | Calx-*β*4～Calx*β*5  Calx-*β*35 | 1b,1c  1b,1a | (Sloan-Heggen et al., 2016) |
| 6 | c.1849G>A(p.Val617Met)  +c.6994A>T(p.Ile2332Phe) | missense  missense | compound heterozygous | Calx-*β*4～Calx-*β*5  Calx-*β*16～Calx-*β*17 | 1b,1c  1b | (Sloan-Heggen et al., 2016) |
| 7 | c.1953A>T(p.Glu651Asp)  +c.8651T>C(p.Val2884Ala) | missense  missense | compound heterozygous | Calx-*β*5  Calx-*β*20 | 1b,1c  1b | (Sloan-Heggen et al., 2016) |
| 8 | c.6086C>T(p.Pro2029Leu)  +c.16472G>A(p.Ser5491Asn) | missense  missense | compound heterozygous | Calx-*β*14  Calx-*β*35～GPS | 1b,1c  1b,1a | (Sloan-Heggen et al., 2016) |
| 9 | c.8401G>A(p.Gly2801Arg)  + c.17108G>A(p.Arg5703His) | missense  missense | compound heterozygous | Calx-*β*19～Calx-*β*20  Calx-*β*35～GPS | 1b  1b,1a | (Sloan-Heggen et al., 2016) |
| 10 | c.8585A>G(p.Tyr2862Cys)  + c.17735C>G(p.Ser5912Cys) | missense  missense | compound heterozygous | Calx-*β*20  E | 1b  1b,1a | (Sloan-Heggen et al., 2016) |
| 11 | c.12185G>A(p.Arg4062Gln)  +c.15967G>C(p.Glu5323Gln) | missense  missense | compound heterozygous | Calx-*β*27  Calx-*β*34 | 1b  1b,1a | (Ziats et al., 2020) |
| 12 | c.12394C>G(p.Pro4132Ala)  + c.14515C>G(p.Gln4839Glu) | missense  missense | compound heterozygous | Calx-*β*27～Calx-*β*28  Calx-*β*32～Calx-*β*233 | 1b  1b,1a | (Sloan-Heggen et al., 2016) |
| 13 | c.13345G>T(p.Asp4449Tyr)  + c.17992G>A(p.Val5998Met) | missense  missense | compound heterozygous | Calx-*β*30  E | 1b,1a  1b,1a | (Sloan-Heggen et al., 2016) |
| 14 | c.14348delT(p.Ile4783Thrfs*5)  +c.506T>C(p.Leu169Pro) | frameshift  missense | compound heterozygous | -  Calx-*β*2 | 1b,1a  1b,1c | (Sloan-Heggen et al., 2016) |
| 15 | c.16544delT(p.Leu5515fsX)  +c.1054C>A (p.Pro352Thr) | frameshift  missense | compound heterozygous | -  Calx-*β*3 | 1b,1a  1b,1c | (Bousfiha et al., 2017) |
| 16 | c.10088_10091del4(p.Val3363AspfsX10)  +c.2399G>A(p.Arg800Gln) | frameshift  missense | compound heterozygous | -  Calx-*β*6 | 1b  1b,1c | (Yang et al., 2013) |
| 17 | c.10213C>T(p.Arg3405X)  +c.14366G>A(p.Arg4789Gln) | nonsense  missense | compound heterozygous | -  Calx-*β*32～Calx-*β*33 | 1b  1b,1a | (Chen et al., 2013) |
| 18 | c.1379delA(p.Ala462ArgfsX36)  +c.4878-4879ins10(p.Ala1630IlefsX1) | frameshift  frameshift | compound heterozygous | -  - | 1b,1c  1b,1c | (Yang et al., 2013) |
| 19 | c.16114G>T(p.Glu5372X) | nonsense | heterozygous | - | 1b,1a | (Yuan et al., 2020) |
| 20 | c.16129G>T(p.Gly5377X) | nonsense | heterozygous | - | 1b,1a | (Yuan et al., 2020) |
| 21 | c.17200G>T(p.Glu5734X) | nonsense | heterozygous | - | 1b,1a | (Yuan et al., 2020) |
| 22 | c.1055C>T(p.Pro352Leu) | missense | heterozygous | Calx-*β*4 | 1b,1c | (Richard et al., 2019) |
| 23 | c.13996A>G(p.Ile4666Val) | missense | heterozygous | Calx-*β*32 | 1b,1a | (Miyagawa et al., 2013) |
|  |  |  |  |  |  |  |
| **Retinitis pigmentosa** | | | | | |  |
| 1 | c.929G>A(p.Gly310Glu)  + c.18601A>C(p.Asn6201His) | missense  missense | compound heterozygous | Calx-*β*3  E | 1b,1c  1b,1a | (Wang et al., 2018) |
| 2 | c.3343G>A(p.Gly1148Asp)  +c.8226T>G(p.Ile2742Met) | missense  missense | compound heterozygous | Calx-*β*9  Calx-*β*19 | 1b,1c  1b | (Wang et al., 2014) |
| 3 | c.7176C>T(p.Ser2392Ser)  + c.13757A>T(p.Glu4586Val) | missense  missense | compound heterozygous | Calx-*β*16～Calx-*β*17  Calx-*β*31 | 1b  1b,1a | (Bravo-Gil et al., 2017) |
| 4 | c.8407G>A(p.Ala2803Thr)  +c.12269C>A(p.Thr4090Ala) | missense  missense | compound heterozygous | Calx-*β*20  Calx-*β*27 | 1b,1c  1b | (Neveling et al., 2013) |
|  |  |  |  |  |  |  |
| **Epilepsy** | | | | | |  |
| 1 | c.43T>A(p.Leu15Ile)  +c.8306T>C(p.Leu2769Ser) | missense  missense | compound heterozygous | Signal peptide  Calx-*β*19 | 1b,1c  1b |  |
| 2 | c.814G>A(p.Val272Ile)  c.9083A>G(p.Asn3028Ser) | missense  missense | compound heterozygous | Calx-*β*2  Calx-*β*21 | 1b,1c  1b | (Liu et al., 2022) |
| 3 | c.10970C>T(p.Ala3657Val)  c.13255A>G (p.Met4419Val) | missense  missense | compound heterozygous | Calx-*β*24  Calx-*β*30 | 1b  1b,1a | (Liu et al., 2022) |
| 4 | c.530C>G(p.Thr177Ser) **^†^** | missense | heterozygous | Calx-*β*2 | 1b,1c | (Myers et al., 2018) |
| 5 | c.1718G>T(p.Gly573Val) | missense | heterozygous | - | 1b,1c | (Dahawi et al., 2021) |
| 6 | c.1970A>G(p.Asn657Ser) | missense | heterozygous | Calx-*β*5 | 1b,1c |  |
| 7 | c.2039A>G(p.Asp680Gly) | missense | heterozygous | Calx-*β*5 | 1b,1c | (Han et al., 2020) |
| 8 | c.3268A>G(p.Ile1090Val) | missense | heterozygous | Calx-*β*8 | 1b,1c | (Myers et al., 2018) |
| 9 | c.3509A>C(p.Tyr1170Ser) | missense | heterozygous | Calx-*β9* | 1b,1c | (Dahawi et al., 2021) |
| 10 | c.5857A>C(p.Ser1953Arg) | missense | heterozygous | Calx-*β*13~ Calx-*β*14 | 1b,1c | (Myers et al., 2018) |
| 11 | c.7342G>A(p.Ala2448Thr) | missense | heterozygous | Calx-*β*17 | 1b | (Myers et al., 2018) |
| 12 | c.8290T>C(p.Ser2764Pro) | missense | heterozygous | Calx-*β*19 | 1b | (Nakayama et al., 2002) |
| 13 | c.8068A>G(p.Thr2690Ala) | missense | heterozygous | Calx-*β*19 | 1b |  |
| 14 | c.9069G>C(p.Arg3023Ser) | missense | heterozygous | Calx-*β*21 | 1b |  |
| 15 | c.9214G>A(p.Gly3072Ser) | missense | heterozygous | Calx-*β*22 | 1b | (Dahawi et al., 2021) |
| 16 | c.9459A>G(p.Ile3153Met) | missense | heterozygous | Calx-*β*22 | 1b |  |
| 17 | c.9701C>T(p.Ala3234Val) | missense | heterozygous | EAR4 | 1b |  |
| 18 | c.13616T>A(p.Val4539Glu) | missense | heterozygous | Calx-*β*31 | 1b,1a |  |
| 19 | c.13919G>A(p.Gly4640Glu) | missense | heterozygous | Calx-*β*32 | 1b,1a | (Dahawi et al., 2021) |
| 20 | c.13949C>G(p.Ser4650Cys) | missense | heterozygous | Calx-*β*32 | 1b,1a | (Myers et al., 2018) |
| 21 | c.17195C>T(p.Pro5732Leu) | missense | heterozygous | - | 1b,1a | (Dahawi et al., 2021) |
| 22 | c.7560delA(p.Asn2521IlefsX19) | frameshift | heterozygous | Calx-*β*17 | 1b |  |
| 23 | c.10724_10725insG(p.Ile3575MetfsX2) | frameshift | heterozygous | Calx-*β*23 | 1b |  |
| 24 | c.8495C>A(p.Ser2832X) | nonsense | heterozygous | Calx-*β*22 | 1b | (Nakayama et al., 2002) |

^†^identified in two unrelated cases

Abbreviations: Calx-*β,* calcium exchanger β; E, extracellular domain; EAR, epilepsy-associated repeats; GPS, G protein proteolytic site; Ref., references; TM, transmembrane domain.

**References**

Ammar-Khodja, F., Bonnet, C., Dahmani, M., Ouhab, S., Lefevre, G.M., Ibrahim, H., et al. (2015). Diversity of the causal genes in hearing impaired Algerian individuals identified by whole exome sequencing. *Mol Genet Genomic Med* 3(3)**,** 189-196. doi: 10.1002/mgg3.131.

Aparisi, M.J., Aller, E., Fuster-Garcia, C., Garcia-Garcia, G., Rodrigo, R., Vazquez-Manrique, R.P., et al. (2014). Targeted next generation sequencing for molecular diagnosis of Usher syndrome. *Orphanet J Rare Dis* 9. doi: 10.1186/s13023-014-0168-7.

Baux, D., Vache, C., Blanchet, C., Willems, M., Baudoin, C., Moclyn, M., et al. (2017). Combined genetic approaches yield a 48% diagnostic rate in a large cohort of French hearing-impaired patients. *Sci Rep* 7. doi: 10.1038/s41598-017-16846-9.

Besnard, T., Garcia-Garcia, G., Baux, D., Vache, C., Faugere, V., Larrieu, L., et al. (2014). Experience of targeted Usher exome sequencing as a clinical test. *Mol Genet Genomic Med* 2(1)**,** 30-43. doi: 10.1002/mgg3.25.

Besnard, T., Vache, C., Baux, D., Larrieu, L., Abadie, C., Blanchet, C., et al. (2012). Non-USH2A mutations in USH2 patients. *Hum Mutat* 33(3)**,** 504-510. doi: 10.1002/humu.22004.

Bonnet, C., Grati, M.h., Marlin, S., Levilliers, J., Hardelin, J.-P., Parodi, M., et al. (2011). Complete exon sequencing of all known Usher syndrome genes greatly improves molecular diagnosis. *Orphanet J Rare Dis* 6. doi: 10.1186/1750-1172-6-21.

Bonnet, C., Riahi, Z., Chantot-Bastaraud, S., Smagghe, L., Letexier, M., Marcaillou, C., et al. (2016). An innovative strategy for the molecular diagnosis of Usher syndrome identifies causal biallelic mutations in 93% of European patients. *Eur J Hum Genet* 24(12)**,** 1730-1738. doi: 10.1038/ejhg.2016.99.

Bousfiha, A., Bakhchane, A., Charoute, H., Detsouli, M., Rouba, H., Charif, M., et al. (2017). Novel compound heterozygous mutations in the GPR98 (USH2C) gene identified by whole exome sequencing in a Moroccan deaf family. *Mol Biol Rep* 44(5)**,** 429-434. doi: 10.1007/s11033-017-4129-9.

Bravo-Gil, N., Gonzalez-del Pozo, M., Martin-Sanchez, M., Mendez-Vidal, C., Rodriguez-de la Rua, E., Borrego, S., et al. (2017). Unravelling the genetic basis of simplex Retinitis Pigmentosa cases. *Sci Rep* 7. doi: 10.1038/srep41937.

Bryant, L., Lozynska, O., Maguire, A.M., Aleman, T.S., and Bennett, J. (2018). Prescreening whole exome sequencing results from patients with retinal degeneration for variants in genes associated with retinal degeneration. *Clin Ophthalmol* 12**,** 49-63. doi: 10.2147/opth.S147684.

Chen, X., Zhao, K., Sheng, X., Li, Y., Gao, X., Zhang, X., et al. (2013). Targeted Sequencing of 179 Genes Associated with Hereditary Retinal Dystrophies and 10 Candidate Genes Identifies Novel and Known Mutations in Patients with Various Retinal Diseases. *Invest Ophthalmol Vis Sci* 54(3)**,** 2186-2197. doi: 10.1167/iovs.12-10967.

Dahawi, M., Elmagzoub, M.S., A Ahmed, E., Baldassari, S., Achaz, G., Elmugadam, F.A., et al. (2021). Involvement of Gene in Familial Forms of Genetic Generalized Epilepsy. *Frontiers In Neurology* 12**,** 738272. doi: 10.3389/fneur.2021.738272.

Ebermann, I., Wiesen, M.H.J., Zrenner, E., Lopez, I., Pigeon, R., Kohl, S., et al. (2009). GPR98 mutations cause Usher syndrome type 2 in males. *J Med Genet* 46(4)**,** 277-280. doi: 10.1136/jmg.2008.059626.

Fuster-Garcia, C., Garcia-Garcia, G., Jaijo, T., Fornes, N., Ayuso, C., Fernandez-Burriel, M., et al. (2018). High-throughput sequencing for the molecular diagnosis of Usher syndrome reveals 42 novel mutations and consolidates CEP250 as Usher-like disease causative. *Sci Rep* 8. doi: 10.1038/s41598-018-35085-0.

Garcia-Garcia, G., Besnard, T., Baux, D., Vache, C., Aller, E., Malcolm, S., et al. (2013). The contribution of GPR98 and DFNB31 genes to a Spanish Usher syndrome type 2 cohort. *Mol Vis* 19**,** 367-373.

Gloeckle, N., Kohl, S., Mohr, J., Scheurenbrand, T., Sprecher, A., Weisschuh, N., et al. (2014). Panel-based next generation sequencing as a reliable and efficient technique to detect mutations in unselected patients with retinal dystrophies. *Eur J Hum Genet* 22(1)**,** 99-104. doi: 10.1038/ejhg.2013.72.

Gu, X., Guo, L., Ji, H., Sun, S., Chai, R., Wang, L., et al. (2015). Genetic testing for sporadic hearing loss using targeted massively parallel sequencing identifies 10 novel mutations. *Clin Genet* 87(6)**,** 588-593. doi: 10.1111/cge.12431.

Han, J.Y., Lee, H.J., Lee, Y.-M., and Park, J. (2020). Identification of Missense Mutation as a Candidate Genetic Cause of Familial Febrile Seizure 4. *Children (Basel, Switzerland)* 7(9). doi: 10.3390/children7090144.

Hilgert, N., Kahrizi, K., Dieltjens, N., Bazazzadegan, N., Najmabadi, H., Smith, R.J.H., et al. (2009). A large deletion in GPR98 causes type IIC Usher syndrome in male and female members of an Iranian family. *J Med Genet* 46(4)**,** 272-276. doi: 10.1136/jmg.2008.060947.

Hmani-Aifa, M., Benzina, Z., Zulfiqar, F., Dhouib, H., Shahzadi, A., Ghorbel, A., et al. (2009). Identification of two new mutations in the GPR98 and the PDE6B genes segregating in a Tunisian family. *Eur J Hum Genet* 17(4)**,** 474-482. doi: 10.1038/ejhg.2008.167.

Jiang, L., Liang, X., Li, Y., Wang, J., Zaneveld, J.E., Wang, H., et al. (2015). Comprehensive molecular diagnosis of 67 Chinese Usher syndrome probands: high rate of ethnicity specific mutations in Chinese USH patients. *Orphanet J Rare Dis* 10. doi: 10.1186/s13023-015-0329-3.

Kahrizi, K., Bazazzadegan, N., Jamali, L., Nikzat, N., Kashef, A., and Najmabadi, H. (2014). A novel mutation of the USH2C (GPR98) gene in an Iranian family with Usher syndrome type II. *J Genet* 93(3)**,** 837-841. doi: 10.1007/s12041-014-0443-3.

Krawitz, P.M., Schiska, D., Kruger, U., Appelt, S., Heinrich, V., Parkhomchuk, D., et al. (2014). Screening for single nucleotide variants, small indels and exon deletions with a next-generation sequencing based gene panel approach for Usher syndrome. *Mol Genet Genomic Med* 2(5)**,** 393-401. doi: 10.1002/mgg3.92.

Lenarduzzi, S., Morgan, A., Faletra, F., Cappellani, S., Morgutti, M., Mezzavilla, M., et al. (2019). Next generation sequencing study in a cohort of Italian patients with syndromic hearing loss. *Hear Res* 381. doi: 10.1016/j.heares.2019.07.006.

Liu, Z., Ye, X., Zhang, J., Wu, B., Dong, S., and Gao, P. (2022). Biallelic ADGRV1 variants are associated with Rolandic epilepsy. *Neurological Sciences : Official Journal of the Italian Neurological Society and of the Italian Society of Clinical Neurophysiology* 43(2)**,** 1365-1374. doi: 10.1007/s10072-021-05403-y.

Magliulo, G., Iannella, G., Gagliardi, S., Iozzo, N., Plateroti, R., Mariottini, A., et al. (2017). Usher's Syndrome Type II: A Comparative Study of Genetic Mutations and Vestibular System Evaluation. *Otolaryngol Head Neck Surg* 157(5)**,** 853-860. doi: 10.1177/0194599817715235.

Malm, E., Ponjavic, V., Moller, C., Kimberling, W.J., and Andreasson, S. (2011). Phenotypes in Defined Genotypes Including Siblings with Usher Syndrome. *Ophthalmic Genet* 32(2)**,** 65-74. doi: 10.3109/13816810.2010.536064.

Miyagawa, M., Naito, T., Nishio, S.-y., Kamatani, N., and Usami, S.-i. (2013). Targeted Exon Sequencing Successfully Discovers Rare Causative Genes and Clarifies the Molecular Epidemiology of Japanese Deafness Patients. *PloS One* 8(8). doi: 10.1371/journal.pone.0071381.

Moteki, H., Yoshimura, H., Azaiez, H., Booth, K.T., Shearer, A.E., Sloan, C.M., et al. (2015). USH2 Caused by GPR98 Mutation Diagnosed by Massively Parallel Sequencing in Advance of the Occurrence of Visual Symptoms. *Ann Otol Rhinol Laryngol* 124**,** 123S-128S. doi: 10.1177/0003489415574070.

Myers, K.A., Nasioulas, S., Boys, A., McMahon, J.M., Slater, H., Lockhart, P., et al. (2018). ADGRV1 is implicated in myoclonic epilepsy. *Epilepsia* 59(2)**,** 381-388. doi: 10.1111/epi.13980.

Nakayama, J., Fu, Y.-H., Clark, A.M., Nakahara, S., Hamano, K., Iwasaki, N., et al. (2002). A nonsense mutation of the MASS1 gene in a family with febrile and afebrile seizures. *Ann Neurol* 52(5)**,** 654-657. doi: 10.1002/ana.10347.

Neuhaus, C., Eisenberger, T., Decker, C., Nagl, S., Blank, C., Pfister, M., et al. (2017). Next-generation sequencing reveals the mutational landscape of clinically diagnosed Usher syndrome: copy number variations, phenocopies, a predominant target for translational read-through, and PEX26 mutated in Heimler syndrome. *Mol Genet Genomic Med* 5(5)**,** 531-552. doi: 10.1002/mgg3.312.

Neveling, K., Collin, R.W.J., Gilissen, C., van Huet, R.A.C., Visser, L., Kwint, M.P., et al. (2013). Next Generation Genetic Testing for Retinitis Pigmentosa (vol 33, pg 963, 2012). *Hum Mutat* 34(8)**,** 1181-1181. doi: 10.1002/humu.22357.

Reddy, R., Fahiminiya, S., El Zir, E., Mansour, A., Megarbane, A., Majewski, J., et al. (2014). Molecular Genetics of the Usher Syndrome in Lebanon: Identification of 11 Novel Protein Truncating Mutations by Whole Exome Sequencing. *PLoS One* 9(9). doi: 10.1371/journal.pone.0107326.

Richard, E.M., Santos-Cortez, R.L.P., Faridi, R., Rehman, A.U., Lee, K., Shahzad, M., et al. (2019). Global genetic insight contributed by consanguineous Pakistani families segregating hearing loss. *Hum Mutat* 40(1)**,** 53-72. doi: 10.1002/humu.23666.

Santana, E.E., Fuster-Garcia, C., Aller, E., Jaijo, T., Garcia-Bohorquez, B., Garcia-Garcia, G., et al. (2019). Genetic Screening of the Usher Syndrome in Cuba. *Front Genet* 10. doi: 10.3389/fgene.2019.00501.

Sloan-Heggen, C.M., Bierer, A.O., Shearer, A.E., Kolbe, D.L., Nishimura, C.J., Frees, K.L., et al. (2016). Comprehensive genetic testing in the clinical evaluation of 1119 patients with hearing loss. *Hum Genet* 135(4)**,** 441-450. doi: 10.1007/s00439-016-1648-8.

Stabej, P.L.Q., Saihan, Z., Rangesh, N., Steele-Stallard, H.B., Ambrose, J., Coffey, A., et al. (2012). Comprehensive sequence analysis of nine Usher syndrome genes in the UK National Collaborative Usher Study. *J Med Genet* 49(1)**,** 27-36. doi: 10.1136/jmedgenet-2011-100468.

Sun, T., Xu, K., Zhang, X., and Li, Y. (2018). Comprehensive Molecular Screening in a Large Chinese Cohort with Usher Syndrome. *Invest Ophthalmol Vis Sci* 59(9).

Turro, E., Astle, W.J., Megy, K., Graef, S., Greene, D., Shamardina, O., et al. (2020). Whole-genome sequencing of patients with rare diseases in a national health system. *Nature* 583(7814)**,** 96-+. doi: 10.1038/s41586-020-2434-2.

Wang, F., Wang, H., Tuan, H.-F., Nguyen, D.H., Sun, V., Keser, V., et al. (2014). Next generation sequencing-based molecular diagnosis of retinitis pigmentosa: identification of a novel genotype-phenotype correlation and clinical refinements. *Hum Genet* 133(3)**,** 331-345. doi: 10.1007/s00439-013-1381-5.

Wang, L., Zhang, J., Chen, N., Wang, L., Zhang, F., Ma, Z., et al. (2018). Application of Whole Exome and Targeted Panel Sequencing in the Clinical Molecular Diagnosis of 319 Chinese Families with Inherited Retinal Dystrophy and Comparison Study. *Genes (Basel)* 9(7). doi: 10.3390/genes9070360.

Wei, C., Yang, L., Cheng, J., Imani, S., Fu, S., Lv, H., et al. (2018). A novel homozygous variant of GPR98 causes usher syndrome type IIC in a consanguineous Chinese family by next generation sequencing. *BMC Med Genet* 19. doi: 10.1186/s12881-018-0602-0.

Weston, M.D., Luijendijk, M.W.J., Humphrey, K.D., Moller, C., and Kimberling, W.J. (2004). Mutations in the VLGR1 gene implicate G-protein signaling in the pathogenesis of Usher syndrome type II. *Am J Hum Genet* 74(2)**,** 357-366. doi: 10.1086/381685.

Yang, T., Wei, X., Chai, Y., Li, L., and Wu, H. (2013). Genetic etiology study of the non-syndromic deafness in Chinese Hans by targeted next-generation sequencing. *Orphanet J Rare Dis* 8. doi: 10.1186/1750-1172-8-85.

Yuan, Y., Li, Q., Su, Y., Lin, Q., Gao, X., Liu, H., et al. (2020). Comprehensive genetic testing of Chinese SNHL patients and variants interpretation using ACMG guidelines and ethnically matched normal controls. *Eur J Hum Genet* 28(2)**,** 231-243. doi: 10.1038/s41431-019-0510-6.

Zampaglione, E., Kinde, B., Place, E.M., Navarro-Gomez, D., Maher, M., Jamshidi, F., et al. (2020). Copy-number variation contributes 9% of pathogenicity in the inherited retinal degenerations. *Genet Med* 22(6)**,** 1079-1087. doi: 10.1038/s41436-020-0759-8.

Zhang, N., Wang, J., Liu, S., Liu, M., and Jiang, F. (2018). Identification of two novel compound heterozygous mutations of ADGRV1 in a Chinese family with Usher syndrome type IIC. *Ophthalmic Genet* 39(4)**,** 517-521. doi: 10.1080/13816810.2018.1479430.

Ziats, M.N., Ahmad, A., Bernat, J.A., Fisher, R., Glassford, M., Hannibal, M.C., et al. (2020). Genotype-phenotype analysis of 523 patients by genetics evaluation and clinical exome sequencing. *Pediatr Res* 87(4)**,** 735-739. doi: 10.1038/s41390-019-0611-5.
